# Characterisation of the willow phenylalanine ammonia-lyase (*PAL*) gene family reveals expression differences compared with poplar

**DOI:** 10.1016/j.phytochem.2015.06.005

**Published:** 2015-09

**Authors:** Femke de Jong, Steven J. Hanley, Michael H. Beale, Angela Karp

**Affiliations:** a AgroEcology Department, Rothamsted Research, Harpenden, Hertfordshire AL5 2JQ, United Kingdom; b Plant Biology and Crop Sciences Department, Rothamsted Research, Harpenden, Hertfordshire AL5 2JQ, United Kingdom

**Keywords:** 4CL, 4-coumarate-CoA ligase, C4H, *trans*-cinnamate 4 monooxygenase, KFB, Kelch repeat F-box, PAL, phenylalanine ammonia-lyase, SNP, single-nucleotide polymorphism, YFP, yellow fluorescent protein, l-Phenylalanine ammonia-lyase (PAL), *Salix viminalis* (willow), Subcellular localisation, Enzyme kinetics, Gene expression, Phenylpropanoid metabolism, Gene family

## Abstract

Willow is an important biomass crop for the bioenergy industry, and therefore optimal growth with minimal effects of biotic and abiotic stress is essential. The phenylpropanoid pathway is responsible for the biosynthesis of not only lignin but also of flavonoids, condensed tannins, benzenoids and phenolic glycosides which all have a role in protecting the plant against biotic and abiotic stress. All products of the phenylpropanoid pathway are important for the healthy growth of short rotation cropping species such as willow. However, the phenylpropanoid pathway in willow remains largely uncharacterised. In the current study we identified and characterised five willow phenylalanine ammonia-lyase (PAL) genes, which encode enzymes that catalyse the deamination of l-phenylalanine to form *trans*-cinnamic acid, the entry point into the phenylpropanoid pathway. Willow *PAL1*, *PAL2*, *PAL3* and *PAL4* genes were orthologous to the poplar genes. However no orthologue of *PAL5* appears to be present in willow. Moreover, two tandemly repeated *PAL2* orthologues were identified in a single contig. Willow PALs show similar sub-cellular localisation to the poplar genes. However, the enzyme kinetics and gene expression of the willow PAL genes differed slightly, with willow *PAL2* being more widely expressed than its poplar orthologues implying a wider role for PALs in the production of flavonoids, condensed tannins, benzenoids, and phenolic glycosides, in willow.

## Highlights

• Five *Salix viminalis* (willow) phenylalanine ammonia-lyase (PAL) genes were identified and functionally characterized.
• Willow PALs show similar sub-cellular localisation to the poplar genes.
• Willow PALs show difference in enzyme kinetics and gene expression to the poplar genes.

## Graphical abstract

The willow PAL family consists of 5 genes encoding 4 isozymes that have higher kinetic activity, and a wider gene expression pattern, than that found for the closely related poplar PAL family.

**Figure:**
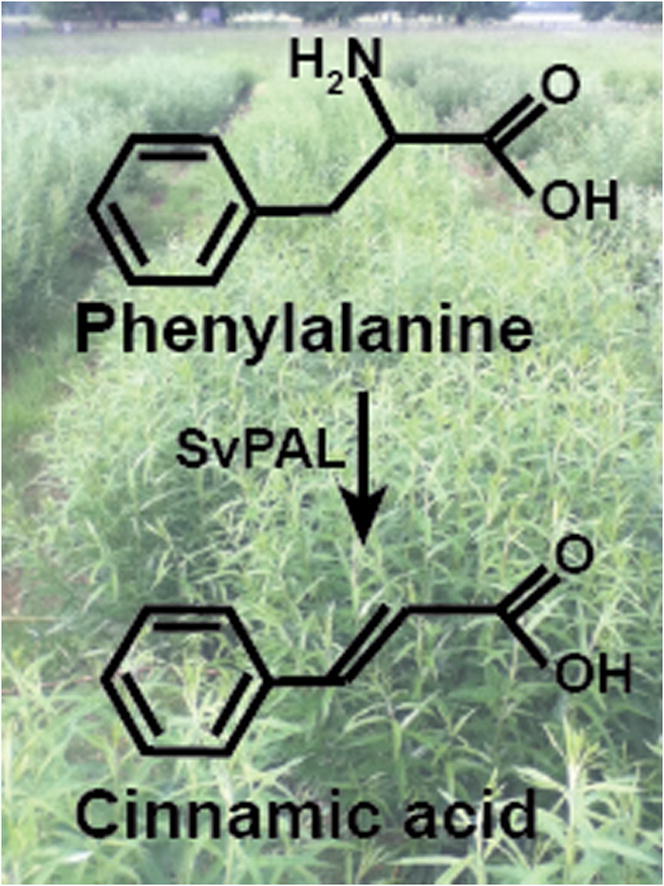

## 1 Introduction

The two main genera that comprise the Salicaceae, *Salix* (willows) and *Populus* (poplars and aspens), produce a wide range of secondary metabolites, of which those of the phenylpropanoid pathway are both abundant and diverse (Boeckler et al., 2011; Tsai et al., 2006). In addition to the promise shown by short rotation coppice willow as a fast-growing, dedicated biomass feedstock alternative to fossil fuels, this wide range of secondary products in this crop has the potential to be exploited by chemical and natural product industries (Karp, 2013; Karp et al., 2011). The phenylpropanoid pathway produces both the well-studied flavonoids, condensed tannins, and lignin, as well as the lesser studied benzenoids and phenolic glycosides (Babst et al., 2010; Boeckler et al., 2011; Shi et al., 2010, 2013; Tsai et al., 2006; Vogt, 2010). The entry point from primary metabolism into phenylpropanoid metabolism is the deamination of l-phenylalanine by phenylalanine ammonia-lyase (PAL, EC.4.3.1.24) to form *trans*-cinnamic acid (Vogt, 2010). PAL enzyme activity determines the flux through the phenylpropanoid pathway and the rate of phenylpropanoid production (Bate et al., 1994; Wang et al., 2014). Therefore, a better understanding of willow PAL expression and activity will aid the breeding and selection of willows for bioenergy and chemical product end-uses.

PAL is regulated developmentally and environmentally by transcriptional regulation through MYB, LIM and NTS transcription factors (Zhao and Dixon, 2011; Zhong and Ye, 2007). In addition, post-transcriptional regulation has been observed. In French bean phosphorylation of recombinant poplar PtrPAL by endogenous PAL-kinase was demonstrated to reduce PtrPAL stability (Allwood et al., 1999). Moreover, it was recently shown that in Arabidopsis, PAL activity is regulated post-transcriptionally through ubiquitination by Kelch repeat F-box (KFB) proteins to target PAL for degradation (Zhang et al., 2013). It has also been proposed that the flux through the phenylpropanoid pathway is regulated via metabolic channelling. For example, in *Nicotiana tabacum*, metabolic channelling towards lignin production occurs, in which NtPAL1 shows coupling with *N. tabacum trans*-cinnamate 4-monooxygenase (NtC4H), which catalyses the next step in the pathway towards lignin (Achnine et al., 2004; Rasmussen and Dixon, 1999).

*PAL* encoding genes are generally well studied and are commonly found as small gene families comprising one to five members (Cochrane et al., 2004; Huang et al., 2010; Rawal et al., 2013; Reichert et al., 2009; Tsai et al., 2006), although in some plants such as in Eucalyptus (Carocha et al., 2015) and watermelon (Dong and Shang, 2013) the *PAL* gene family is larger than five members. The encoded proteins form a homo- or heterotetramer and the different *PAL* genes are thought to be involved in different branches of the phenylpropanoid pathway (Cochrane et al., 2004; Reichert et al., 2009; Tsai et al., 2006), an assumption now confirmed for the poplar *PAL* gene family (Kao et al., 2002; Shi et al., 2013; Tsai et al., 2006).

The *PAL* gene family in poplar (*Populus trichocarpa*) consists of five genes (*PtrPAL1–5*), which are separated into two groups by phylogenetic analysis (Tsai et al., 2006). Members of group A (*PtrPAL2*, *4* and *5*) are mainly expressed in xylem and root tips while group B genes (*PtrPAL1* and *3*) are more widely expressed (Tsai et al., 2006). In poplar this clear difference in expression of the *PAL* genes, combined with the co-localization of 4-coumarate-CoA ligase 2 (Ptr4CL2) and condensed tannins with PtrPAL1 suggests that, PtrPAL1 and 3 are predominantly responsible for the production of condensed tannins, flavonoids and other phenol metabolites, whereas PtrPAL2, 4 and 5 are predominantly responsible for the production of lignin (Kao et al., 2002; Tsai et al., 2006; Shi et al., 2013).

In contrast, in Arabidopsis, which has 4 *PAL* genes, *AtPAL1* and *AtPAL2* are predominantly expressed in most tissues with both *AtPAL3* and *4* expressed at lower levels (Cochrane et al., 2004). Using single, double, triple and quadruple *atpal* mutants, Huang et al. (2010) showed that there is redundancy in the role of individual AtPAL proteins. Given that all AtPAL proteins have a redundant role in the production of lignin and benzenoids, and that both AtPAL1 and 2 were shown to have a redundant role in flavonoid production (Huang et al., 2010), the different roles for individual PALs may not always conform to those suggested from the poplar studies.

Willow is an important biomass crop for the heat and power industries but could also be a potential feedstock for biofuels and other industrial products (Karp, 2013; Karp et al., 2011). Optimal growth is therefore essential. However, trees are perennial with long life cycles and are subject to continual environmental stresses for which they need protection. PAL activity determines the flux through the phenylpropanoid pathway contributing both to the production of lignin for growth, as well as the production of flavonoids, condensed tannins, and phenol glycosides for protection against high UV, visible radiation herbivores and pathogens (Karabourniotis et al., 2014; Tsai et al., 2006). In this respect, it is a key gene of interest with respect to the breeding of willows. To investigate PAL activity in willow we identified five *PAL* genes from the common osier, *Salix viminalis* L., a species frequently used in biomass breeding programmes, through homology searches with poplar *PAL* genes. Subsequently, we analysed, expression patterns, recombinant protein activity and subcellular localisation. Our results show that even though they are closely related to PtrPALs, willow PALs (SvPALs) display some differences in gene regulation and enzyme activity.

## 2 Results

### 2.1 Cloning phenylalanine ammonia lyase

**Fig. 1:**
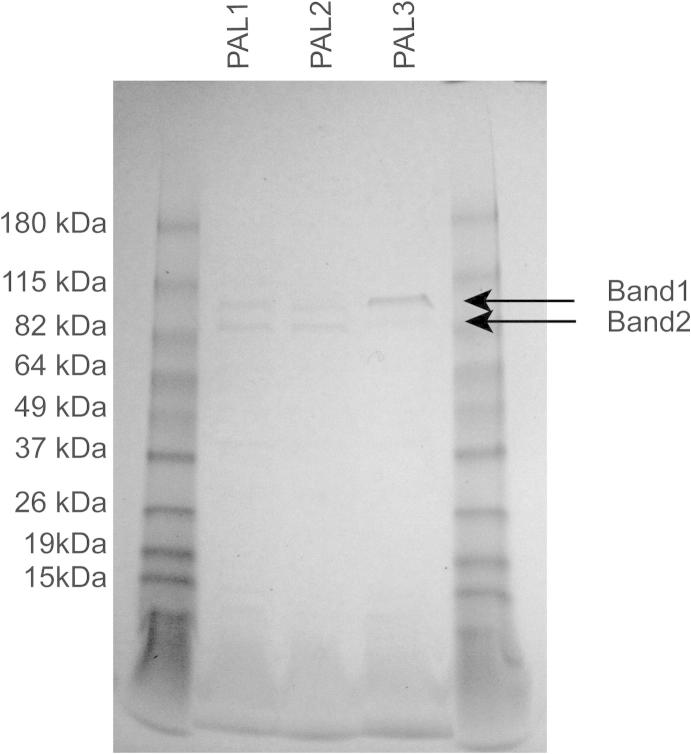
Analysis of recombinant SvPAL by SDS–PAGE. Denatured 6His-SvPAL recombinant proteins were separated on a 4–20% acrylamide SDS–PAGE gel. The marker lane shows the denatured protein molecular weight markers with the size label on the left. For SvPAL1–3 a double band was observed around between the 82 and 115 kDa as indicated by the arrows.

**Fig. 2:**
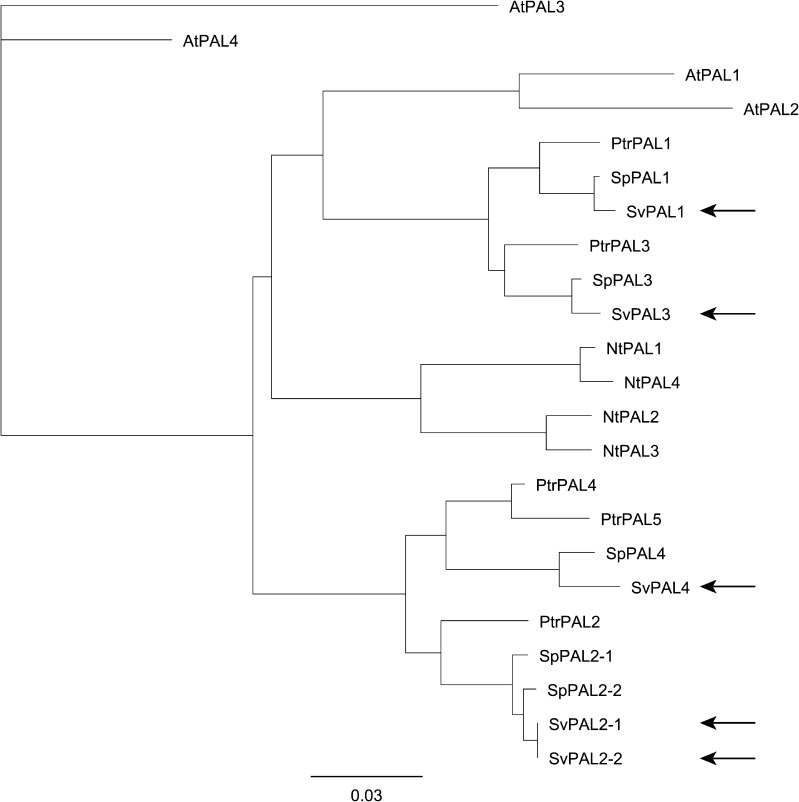
Phylogenetic tree of the phenylalanine ammonia-lyase proteins of Arabidopsis (*Arabidopsis thaliana*, At), Poplar (*Populus trichocarpa*, Ptr), Tobacco (*Nicotiana tabacum*, Nt), and Willow (*Salix viminalis*: Sv, *Salix purpurea*: Sp). AtPAL1: At2G37040, AtPAL2: At3G53260, AtPAL3: At5G04230, AtPAL4: At3G10340, NtPAL1: P25872, NtPAL2: P35513, NtPAL3: P45733, NtPAL4: ACJ66297, PtrPAL1: Potri.006G126800, PtrPAL2: Potri.008G038200, PtrPAL3: Potri.016G091100, PtrPAL4: Potri.010G0224100, PtrPAL5: Potri.010G0224200, SpPAL1: SapurV1A.0141s0150, SpPAL2–1: SapurV1A.1030s0130, SpPAL2–2: SapurV1A.1030s0120, SpPAL3: SapurV1A.0765s0080, SpPAL4: SapurV1A.0518s0200. SvPALs are indicated by the arrows.

Homology searches using *PAL* gene sequences identified putative *S. viminalis* homologues for four of the five poplar *PAL* genes, which were named according to their poplar orthologues, *SvPAL1* for *PtrPAL1* (Potri.006G126800; 95.7% nucleotide identity), *SvPAL2* for *PtrPAL2* (Potri.008G038200; 95.2% identity), *SvPAL3* for *PtrPAL3* (Potri.016G091100; 95.9% identity), and *SvPAL4* for *PtrPAL4* (Potri.010G224100; 93.5% identity). No orthologue was detected for *PtrPAL5* (Potri.010G224200). In addition, two tandemly repeated copies of *SvPAL2* were detected on a single willow contig (100% nucleotide identity) and were named *SvPAL2–1* and *SvPAL2–2.* For all *S. viminalis PAL* genes, orthologues were found in the recently released *Salix purpurea* genome (97–99% homology). Consistent with *S. viminalis*, no putative *S. purpurea* orthologue of *PtrPAL5* was identified. In contrast, to *S. viminalis SvPAL2–1* and *SvPAL2–2*, in *S. purpurea SpPAL2–1* and *SpPAL2–2* do not share 100% nucleotide identity, with one SNP resulting in an early stop codon in SpPAL2–1 amino acid sequence (S-Fig. S2, Online Resource 2). The genes encoding *SvPAL* were between 2160 and 2136 bases long encoding for proteins between 711 and 719 amino acids with calculated molecular weights between 77.6 and 77.9 kDa and calculated pI values ranging between 6.03 and 6.56 (Table 1). To confirm the calculated molecular weight an SDS–PAGE gel analysis was done with purified recombinant 6His-SvPAL protein, showing two bands of around 90 and 76 kDa, respectively (Fig. 1), likely responding to 6His-SvPAL (band 1) and SvPAL (band 2). SvPALs shared between 70–84% amino acid sequence identity with those from Arabidopsis and 81–85% amino acid sequence identity with tobacco (*N. tabacum*). Phylogenetic analysis of the corresponding amino acid sequences showed that the SvPALs cluster together with SpPALs and PtrPALs in two distinct groups (Fig. 2), the first (group B) comprising Sv/Sp/PtrPAL 1 and 3, along with AtPAL1 and AtPAL3 and the second (group A) comprising Sv/Sp/PtrPAL2 and 4 and PtrPAL5.

**Fig. 3:**
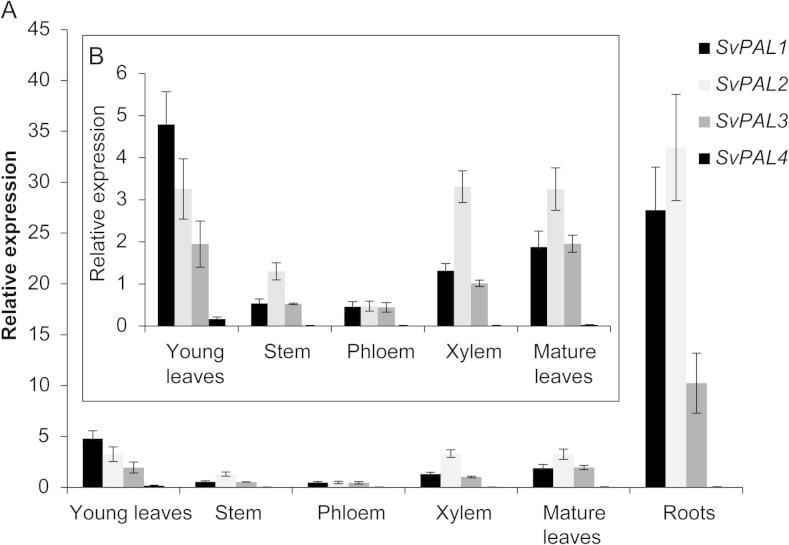
qRT-PCR analysis of the level of expression of *SvPAL1*, *SvPAL2*, *SvPAL3* and *SvPAL4* in A) willow young leaves, stem, phloem, xylem, mature leaves and root tissue and B) without the root data included. Gene expression values were normalized against the willow Sv*TIP4-like* gene. The values are the means of three replicates ± SD.

To further compare the poplar and willow *PAL* genes, the expression of the *SvPAL* genes was analysed in young leaves, stem, phloem, xylem, mature fully expanded leaves and roots. Due to the 100% nucleotide identity of *SvPAL2–1* and *SvPAL2–2*, gene expression of these two genes could not be analysed separately. As shown in Fig. 3, qRT-PCR analysis showed that *SvPAL1*, *2*, and 3 are highly expressed in roots and to a lesser extent in young and mature leaves. *SvPAL1*, *2* and *3* were expressed at a similar, low level in phloem tissue. However in xylem and stem tissue *SvPAL2* gene expression was roughly twice that of for *SvPAL1* and *3*. Expression of *SvPAL4* was much lower than the other willow PAL genes.

To confirm PAL kinetic activity and subcellular localisation, full-length *SvPALs* were cloned using primers designed to homologous willow sequences. *SvPAL1*, *2* and *3* were successfully cloned from cDNA of willow shoot-tip (comprising leaves and stem). As *SvPAL4* expression was only detected in young leaves, cDNA from willow young leaves was generated for cloning *SvPAL4*. However, despite multiple attempts, no full length PCR product could be obtained for *SvPAL4*, suggesting prohibitively low levels of expression for cloning under the experimental conditions used here.

### 2.2 Kinetic characterisation SvPAL

For SvPAL kinetic activity analysis, recombinant 6His-PAL was expressed in *Escherichia coli* BL21 cells and purified with His-Dynabeads®. Recombinant SvPAL1, SvPAL2 and SvPAL3 showed similar catalytic properties (Table 1). Kinetic properties were determined at optimal pH (pH 8.8 for PAL1 and 2 and pH 9.0 for PAL4) and temperature using l-phenylalanine as a substrate. All PALs exhibited standard Michaelis–Menten kinetics with a *K_m_* of 81.6 ± 0.30, 32.35 ± 0.50, and 88.41 ± 0.55 μM and a *V*_max_ of 15.5 ± 0.43, 7.13 ± 0.43, 27.16 ± 1.32 pkat/μg protein for SvPAL1, SvPAL2 and SvPAL3 respectively.

In addition to l-phenylalanine, PAL is known to accept tyrosine, albeit poorly, as a substrate (Langer et al., 2001). Following incubation of SvPALs with l-tyrosine, the formation of *p*-coumaric acid was observed although the activity of all three tested SvPAL enzymes was too low to determine the kinetic parameters. To confirm that there is no TAL activity in willow leaves a crude leaf protein extraction was done to determine native SvPAL activity towards both l-Phe and l-Tyr. Kinetic analysis showed that while l-Phe was converted into t-cinnamic acid at a rate of 2.343 ± 1.006 nmol^−1^ min^−1^ mg protein, no activity with l-Tyr was detected (Table 1).

### 2.3 Subcellular localisation

**Fig. 4:**
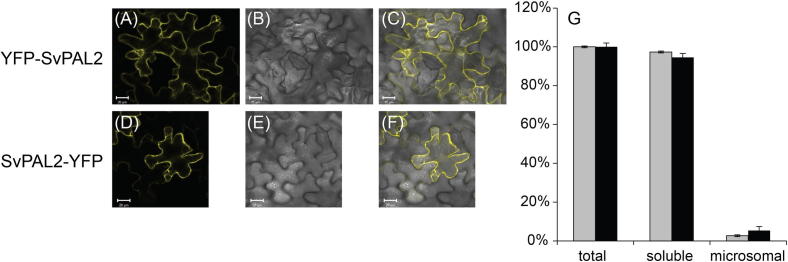
Subcellular localisation of SvPAL2. (A)–(F) An overview of the YFP fusion constructs is shown on the left, with the corresponding transient expression in tobacco epidermal cells is shown on the right. YFP fluorescence is shown in yellow (A, and D). Transmission images of the tobacco epidermal cells are shown in the middle (B, and E) and the overlay of the YFP fluorescence and the transmission images is shown at the far right (C, and F). Bar is 20 μm. (G) Grey bars represent PAL activity in extracts of leaf total, soluble and microsomal protein, scale was set to 100% for total protein activity. Black bars represent percentage of purified total (100%), soluble and microsomal protein. The values are the means of eight replicates ± SE.

Based on the literature PAL is predicted to be cytosolic, although in tobacco NtPAL1 can also be localised at the endoplasmic reticulum (ER) (Achnine et al., 2004). Subcellular localisations of all SvPALs were successfully analysed by transient expression of *SvPAL-YFP* and *YFP-SvPAL* constructs in tobacco leaves. YFP fluorescence was observed in the cytosol of transfected tobacco leaf epidermis cells for all SvPAL-YFP and YFP-SvPAL constructs (Fig. 4 and S-Fig. S3, Online Resource 3). However, when SvPAL-YFP and YFP-SvPAL were overexpressed, ER and/or peroxisomal localisation were also observed. To investigate further possible PAL ER localization, PAL kinetic analysis was done with leaf soluble and microsomal protein fractions. 5% of total PAL activity was observed in the microsomal fraction, with 95% activity detected in the soluble fraction (Fig. 4). With the microsomal protein being less than 3% of the total protein, this shows that there is a considerable amount of PAL activity associated with membranes.

## 3 Discussion

Here we report the identification, cloning and initial characterisation of willow *PAL* genes. PAL catalyses the deamination of l-phenylalanine into *trans*-cinnamic acid and as such commits carbon flux from primary metabolism into phenylpropanoid metabolism. The product of PAL activity, *trans*-cinnamic acid is incorporated into lignin, flavonoids, benzenoids and phenolic glycosides, and thus these enzymes are fundamental in both in the development of wood and the chemical ecology associated with secondary products (Babst et al., 2010; Shi et al., 2010, 2013; Tsai et al., 2006; Vogt, 2010). Although substantial analysis of PAL genes in other organisms has been reported, our results highlight the importance of analysing this gene family in a particular target species. In particular, *Populu*s and *Salix* are sister genera but whilst the SvPAL (*Salix*) sequences are highly homologous to their PtrPAL (*Populus*) orthologues, the measured kinetic parameters of SvPAL are best comparable with those of tobacco and Arabidopsis which exhibit a similar range of specific activity (*V*_max_: SvPAL: 7.3–27.2 nkat/mg, NtPAL: 9.8–19.6 nkat/mg (Reichert et al., 2009), AtPAL: 0.4–10.5 nkat/mg (Cochrane et al., 2004)) and Michaelis–Menten constant (*K_m_*: SvPAL: 32.3–88.5 μM, NtPAL: 36.4–59.8 μM (Reichert et al., 2009), AtPAL: 64–2560 μM (Cochrane et al., 2004)). In contrast, PtrPAL1–5 have a much lower specific activity (*V*_max_: 1–1.5 nkat/mg) and a Michaelis–Menten constant which is at the lower range of the willow *K_m_* values (*K_m_* PtrPAL: 21.3–32.6 μM) (Shi et al., 2013). The turnover number (*k*_cat_) for SvPAL (2.3–29.0 s^−1^), on the other hand, is an order of magnitude higher than those reported for most other PALs (NtPAL: 0.8–1.5 s^−1^ (Reichert et al., 2009), PtrPAL: 1.1–1.4 s^−1^ (Shi et al., 2013), AtPAL 0.1–3.2 s^−1^ (Cochrane et al., 2004)), with the exception of maize (10.6 s^−1^) (Rosler et al., 1997). These data show that, despite close sequence identities of PtrPALs and SvPALs, SvPAL activity appears notably higher in comparison, although, it should be borne in mind that activities can be affected by purification methods. PAL enzyme activity in crude willow leaf protein extracts was within the range of PAL activity in crude poplar SDX protein extracts, suggesting that the *in planta* differences may be less pronounced.

Consistent with the PtrPALs, SvPALs showed cytosolic subcellular localisation. However, when over expressed, all SvPALs tested here showed ER or peroxisomal localisation, in addition to cytosolic localisation. Analysis of PAL activity in soluble and microsome protein fractions indicated considerable PAL activity in the microsome fraction, supporting the suggestion of ER-localization of SvPAL proteins. This phenomenon has been reported previously for tobacco NtPAL1 where Achnine et al. (2004) showed that NtPAL1 shows strong cytosolic and weak ER localisation. However, when co-expressed with *NtC4H*, NtPAL1 localisation shifted completely to the ER, suggesting channelling of *trans*-cinnamic acid between NtPAL1 and NtC4H. No such shift in localization was observed for NtPAL2 (Achnine et al., 2004). The likely ER localisation of SvPAL suggest that a similar process might occur in willow, although, a more detailed study will be needed to confirm this.

Phylogenetic analysis showed that, like *SpPALs* and *PtrPALs*, *SvPAL* genes cluster in two separate clades. An ancient (eurosid) genome-wide duplication (Tsai et al., 2006) is responsible for the formation of the two distinct phylogenetic groups, and the more recent salicoid gene duplication (Tuskan et al., 2006) gave rise to duplication of Sv/Sp/PtrPALs within subclades (*SvPAL1* and *3* in group B and *SvPAL2* and *4* in group A). More recently, tandem duplications have occurred independently in group A. Furthermore, phylogenetic analysis indicated that willow and poplar PALs of group B are more similar to tobacco and Arabidopsis PALs compared to other willow/poplar PALs in group A, suggesting that group A, in which PALs are proposed to have a role in lignin formation, might have conserved functionality for woody species. For group B, it can be proposed that the genes have a role in flavonoid/condensed tannins and benzenoid production.

As the entry point into the phenylpropanoid pathway, PAL is tightly regulated at both expression and post-transcriptional levels. Our analysis of *PAL* gene expression in willow shows that *SvPAL1*, *2* and *3* are highly expressed in roots and leaves, with *SvPAL2* also showing a high expression in xylem, whereas *SvPAL4* was expressed at very low levels in roots and young leaves and at even lower levels in stem, phloem and xylem. This does not completely match the reported gene expression patterns of *PtrPAL*s where expression appears to follow the phylogenetic groups, with group B (*PtrPAL1* and *3*) highly expressed in leaves, roots and xylem and at low levels in stems, and group A (*PtrPAL2*, *4* and *5*), highly expressed in xylem and roots – tissues that are rich in lignin (Tsai et al., 2006). As previously noted, the PAL-group A expression pattern suggests that they are predominantly involved in the production of lignin, while PAL-group B co-expression with Ptr4CL2 and condensed tannin production suggest that they are predominantly involved in the production of condensed tannins, flavonoids and other phenol metabolites (Kao et al., 2002; Shi et al., 2010, 2013; Tsai et al., 2006). Our preliminary expression analysis supports the idea that SvPAL1, 3 and 4 have similar roles to their poplar orthologues. In contrast, willow *SvPAL2* is more widely expressed than *PtrPAL2*. Although *SvPAL2* and *PtrPAL2* are expressed differently, the high amino acid sequence similarity suggests conserved functionality. The low level of *SvPAL4* expression was unexpected and the *SvPAL4* genomic sequence did not reveal anything that might explain its low expression. It is known however that, especially in organisms with large PAL families, gene expression of some of the *PAL* genes is restricted to specific organs such as flowers and fruits (Carocha et al., 2015; Dong and Shang, 2013). Additionally, both *AtPAL* and *OsPAL* have been shown to be induced by UV-B (Huang et al., 2010; Sarma and Sharma, 1999), with *AtPAL* expression also being influenced by temperature, draught and nitrogen levels (Huang et al., 2010; Olsen et al., 2008). Furthermore, *PtrPAL* expression is up-regulated by wounding (Tsai et al., 2006). It is therefore possible that *SvPAL4* is expressed in organs or under conditions not analysed in this paper.

Relatively recent tandem duplications within group A gave rise to *Sv/SpPAL2–1* and *Sv/SpPAL2–2* in willow and *PtrPAL4* and *PtrPAL5* in poplar. Unlike the PAL tandem duplications in *S. purpurea* and *P. trichocarpa* the two *SvPAL2* genes appear to have retained functionality. In contrast, in *S. purpurea* only *SpPAL2–2* appears to be fully functional as *SpPAL2–1* has an early stop codon in its amino acid sequence (S-Fig. S2, Online Resource 2) that is likely to affect its function.

The amino acid sequence of PALs can be divided into five domains (Ritter and Schulz, 2004), a mobile N-terminal domain, which may be involved in interactions with other cellular compounds (residues 1–26, numbering according to SvPAL1), a MIO (4-methylidene-imidazole-5-one) active domain (residues 27–264), a core domain (residues 265–530), a mobile shielding domain with a phosphorylation site that when phosphorylated might limit its movements (residues 531–652), and a C-terminal domain (residues 652–719) (Supplemental Fig. 2). The MIO active domain not only contains the catalytic active MIO group formed by Ala-Ser-Gly, but also a conserved Tyr110. The substitution of Tyr110Phe resulted in an enzyme that is 75000 times reduced in activity (Röther et al., 2002) and the Tyr110 was subsequently shown to be required for correct enzyme conformation (Pilbák et al., 2006). In *P. trichocarpa* amino acid sequence comparison showed that in PtrPAL5 the essential tyrosine 110-loop is altered with a Tyr110His substitution in addition to the deletion of the 4 preceding amino acids (S-Fig. S2, Online Resource 2). Furthermore PtrPAL5 also has an Ala substitution at Thr545, the phosphorylation site in the shielding domain. This suggests that PtrPAL5 amino acid conformation is considerably altered, which likely affects its activity, although PtrPAL5 enzyme activity has been reported previously (Shi et al., 2013). Subsequent analysis of the initial reported PtrPAL5 sequence (EU603320) indicated that this sequence was more similar to PtrPAL4 (2 amino acid difference) than to the PtrPAL5 (19 amino acid difference) sequence in the Phytozome database (http://phytozome.jgi.doe.gov/pz/portal.html), suggesting that, EU6033200 is an allele of PtrPAL4 and not PtrPAL5.

### 3.1 Concluding remarks

In conclusion we have shown that SvPAL genes, although closely related to PtrPALs, have increased activity compared to PtrPAL, and a slightly different gene expression profile. This suggests a higher flux through the phenylpropanoid pathway, which likely has influence on willow lignin content, and production of flavonoids, condensed tannins, and phenol glycosides. This study helps further our understanding of how the phenylpropanoid pathway and PAL are regulated in willow, aiding the breeding of willow crop for biofuel feedstock and industrial product industries. Our findings suggest that it would be worthwhile to study further PAL regulation and activity in relation to the wide variation in concentrations of benzenoids and flavanoids that are known to exist in willows and poplars.

## 4 Materials and methods

### 4.1 General Experimental Procedures

l-phenylalanine (⩾99.0%, CAS # 63-91-2), HPLC grade d-phenylalanine (⩾98%, CAS # 673-06-3), HPLC grade *p*-coumaric acid (⩾98.0%, CAS # 501-98-4), *trans*-cinnamic acid (⩾99%, CAS # 140-10-3), and l-tyrosine (⩾99.0% CAS # 60-18-4) were purchased from Sigma–Aldrich.

### 4.2 Biological material

*S. viminalis* (L.) genotype NWC0663 ‘Pulchra Ruberrima’ was used in all experiments. RNA was extracted from stem and leaf tissue collected on 27 June 2013, following coppicing in the preceding winter For qRT-PCR analysis of *SvPAL* young just emerging leaves, stem, phloem and xylem tissue 30 cm below the shoot tip were taken from field grown willows on 7-4-15, the willows were not coppiced during the previous winter. Mature leaf and root tissue for qRT-PCR were taken from glasshouse grown plants at 22 °C, with 12 h day length. *N. tabacum* cv. Petit Havana plants were grown in glasshouses at 21 °C and with 14 h day length. For all cloning *E. coli* DH5α (Life Technologies) cells were used.

### 4.3 SvPAL identification, cloning and phylogenetic analysis

To identify *SvPAL* genomic sequences, Poplar PAL genes *PtrPAL1* (Potri.006G126800), *PtrPAL2* (Potri.008G038200), *PtrPAL3* (Potri.016G091100), *PtrPAL4* (Potri.010G224100), and *PtrPAL5* (Potri.010G224200) were used to search an in-house preliminary assembly of willow *S. viminalis* NWC0663 genomic sequence (unpublished data) for PAL sequence homologues. The willow PAL genes (accessions: KP728113, KP728114, KP728115 and KP728116) were named according to their poplar orthologue. The sequences were later compared to the PAL gene sequences in the recently released *S. purpurea* genome (*S. purpurea* v1.0, DOE-JGI, http://phytozome.jgi.doe.gov/pz/portal.html#!info?alias=Org_Spurpurea).

*SvPAL* sequences were used for designing primers to obtain full length *PAL* CDS and full length *PAL* CDS without the stop codon (see Table 2 for primer sequences). The *PAL* cDNA sequences were amplified from total willow cDNA by Phusion™ Hot start High-Fidelity DNA Polymerase (New England Biolabs), cloned into the TOPO/D pENTR Gateway® vector (Life Technologies) and completely sequenced. *PAL* identity was confirmed using BLASTN (blast.ncbi.nlm.nih.gov/). These constructs were then used to clone the predicted *PAL* sequences into pDEST17 (Life Technologies), pH7WGY2 and pH7YWG2 (VIB Gent) vectors.

Phylogenetic analysis was conducted using *S. viminalis* and *S. purpurea* (SpPAL1: SapurV1A.0141s0150, SpPAL2–1: SapurV1A.1030s0130, SpPAL2–2: SapurV1A.1030s0120, SpPAL3: SapurV1A.0765s0080, SpPAL4: SapurV1A.0518s0200), *P. trichocarpa* (PtrPAL1: Potri.006G126800, PtrPAL2: Potri.008G038200, PtrPAL3: Potri.016G091100, PtrPAL4: Potri.010G0224100, PtrPAL5: Potri.010G0224200), *Arabidopsis thaliana* (AtPAL1: At2G37040, AtPAL2: At3G53260, AtPAL3: At5G04230, AtPAL4: At3G10340) and *N. tabacum* (NtPAL1: P25872, NtPAL2: P35513, NtPAL3: P45733, NtPAL4: ACJ66297) amino acid sequences using the Neighbor-Joining tree build method within Geneious software (Biomatters).

### 4.4 qRT-PCR analysis

For qRT-PCR analysis of *SvPAL* young just emerging leaves, stem, phloem and xylem tissue 30 cm below the shoot tip were taken from field grown willows on 7-4-15, while roots and mature fully expanded leaves were taken form pot grown willow. RNA extraction was performed as described by Chang et al. (1993) and total cDNA synthesised using a High Capacity cDNA Reverse Transcription Kit (Life Technologies) according to the manufacturer’s instructions. Primers for qRT-PCR were designed to obtain 100–150 bp products Primer sequences are provided in Table 2 and the location of these within each *SvPAL* gene is indicated in S-Fig. S1 (Online Resource 1). PCR was performed using 40 ng of cDNA on a 7500 Fast Real-Time PCR system (Life Technologies) using the SYBR Select Master Mix Kit (Life Technologies) according the manufacturer’s protocol. PCR conditions were: 2 min at 50 °C and 2 min at 95 °C; 40 cycles of 3 s 95 °C, and 30 s 60 °C followed by a melt curve stage: 15 s at 95 °C, 1 min at 60 °C, 0.11 °C/s increase to 95 °C; 15 s 60 °C. Relative transcript levels were calculated by the comparative threshold cycle method (Schmittgen and Livak, 2008) with the willow orthologue (*SvTIP4-like*) of the previously used (Pettengill et al., 2012) poplar *TIP4-like* gene (Potri.009G093200) used as a reference. Expression levels were calculated as the means of two biological replicates, each with three technical replicates ± SD.

### 4.5 Recombinant PAL purification and plant protein purification

For heterologous expression in *E. coli* pDEST17-PAL plasmids were moved to *E. coli* BL21-AI (Life Technologies) cells for recombinant protein expression. A single colony from each transformed *E. coli* BL21 cell line was incubated overnight at 37 °C in 10 ml LB containing ampicillin at a concentration of 100 μg/ml. Inocula were then individually added to 200 ml LB, containing 100 μg/ml ampicillin and grown at 37 °C to an OD_600_ between 0.5 and 0.9 before adding 0.3 ml 20% arabinose to induce *PAL* expression. The cultures were grown for at 37 °C for 4 h before harvesting in 50 ml aliquots by centrifugation at 3000 g for 20 min at 4 °C and stored at −20 °C prior to use. The pellets were thawed and resuspended in 1 ml wash buffer (50 mM Na_2_HPO_4_, 300 mM NaCl, 20 mM imidazole, 1× protease inhibitor cocktail (Sigma)) and 150 μl 200 mM PMSF then sonicated for 6 cycles of 15 s (with 1 min intervals) at 4 °C. Cell debris was removed by centrifugation for 20 min at 12,000 g at 4 °C. The supernatant was added to 100 μl His-Dynabeads® (Life Technologies) and incubated for 15 min under constant rotation at 4 °C. Beads were washed six times with 0.5 ml wash buffer and PAL eluted from the beads by incubation with 2× 200 μl His-elution buffer (50 mM Na_2_HPO_4_, 300 mM NaCl, 300 mM imidazole, 0.01% Tween, 1× protease inhibitor cocktail (Sigma)). The protein was further purified by concentrating the sample using an Amicon Ultra-0.5 Centrifugal Filter Device with a 50,000 NMWL (Millipore).

For extraction of total protein the tissue was ground in liquid nitrogen and 1 ml of extraction buffer (50 mM Tris pH 8.0, 500 mM sucrose, 10% glycerol, 20 mM EDTA, 20 mM EGTA, 50 mM NaF, 0.6% polyvinylpyrrolidone, 10 mM ascorbic acid adjusted to pH 8 with 1 M MES, 5 mM dithiothreitol and 1× protease inhibitor cocktail (Sigma)) was added to 300 mg of tissue. The samples were homogenised and incubated 10 min on ice before centrifuging 2× at 3000 g to remove the debris. The supernatant was used in the PAL kinetic assays.

Extraction of soluble and microsomal proteins was performed according Laugier et al. (2012) in brief, tissue was ground in liquid nitrogen and 1 ml of extraction buffer (50 mM Tris pH 8.0, 500 mM sucrose, 10% glycerol, 20 mM EDTA, 20 mM EGTA, 50 mM NaF, 0.6% polyvinylpyrrolidone, 10 mM ascorbic acid adjusted to pH 8 with 1 M MES, 5 mM dithiothreitol and 1× protease inhibitor cocktail (Sigma)) was added to 300 mg of tissue. Samples were homogenised and incubated for 10 min on ice before centrifuging 2 min at 2000 g. The supernatant was subsequently centrifuged 12 min at 9000 g and 100 min at 16,000 g. The supernatant was used as the soluble protein fraction. The pellet, containing the membrane proteins was resuspended into 100 μl microsomal buffer (10 mM Tris pH 8.0, 300 mM sucrose, 9 mM KCl, 1.5 mM 2-mercaptoethanol) and used as the microsomal fraction.

The concentration was determined by Bradford assay and the purified protein was used directly in the kinetic assays.

### 4.6 SDS–PAGE

Denaturated proteins were analysed on a 4–20% Mini-PROTEAN® TGX™ precast polyacrylamide gel (BioRad) and run on a Mini-PROTEAN Tetra Cell (BioRad), gels were stained with Simply Blue (Life Technologies) to visualise the protein bands.

### 4.7 PAL kinetic assays

For optimal assay conditions the pH and temperature optima were individually determined for each PAL protein. To determine pH optima, assays were performed at 30 °C while varying the pH between pH 6 and 10. The temperature optima were determined at the optimal pH of 8.8 for SvPAL2 and 1 and pH 9 for SvPAL3 while varying the temperature (30–45 °C). Each assay (250 μl total volume) contained 50 mM Tris–HCl (pH 6.0–9.0) or 50 mM borate buffer (pH 10.0), 0.015–2.5 mM l-Phe and 0.5–2 μg of purified PAL. Assays were run for 30 min and UV absorption measurements, relating to the amount *trans*-cinnamic acid formed, were taken at 290 nm every 30 s. For Tyr substrate, assays were run as above, measuring *p*-coumaric acid product at 310 nm. To determine the optimal pH and temperature a Hanes plot was used to calculate the *K_m_* and *V*_max_ after 10 min of reaction and the optimal pH and temperature were those with the highest *V*_max_. To determine the *K_m_* and *V*_max_ for l-Phe and l-Tyr all assays were carried out at 40 °C and at either pH 8.8 (SvPAL1 and 2) or pH 9.0 (SvPAL3), 7 concentrations of l-Phe (0.1–2.5 mM) and l-Tyr (0.02–2 mM). *K_m_* and *V*_max_ were calculated after 10 min incubation according to Cornish-Bowden (2012), using equations for multiplicative error.

### 4.8 Tobacco infiltration and subcellular localisation analysis by confocal microscopy

Six weeks old tobacco (*N. tabacum* cv. Petit Havana) plants were infiltrated with *Agrobacterium tumefaciens* strain GV3101 transformed with one of the following constructs, pH7WGY2-SvPAL1, pH7WGY2-SvPAL2, pH7WGY2-SvPAL3, pH7YWG2-SvPAL2, pH7YWG2-SvPAL3, or pH7YWG2-SvPAL4 as described by Sparkes et al. (2006). Agrobacterium was grown for 48 h before pelleting 1 ml of culture, the pellet was washed in infiltration media (0.5% (w/v) d-glucose, 50 mM MES, 2 mM Na_3_PO_4_, 0.1 mM acetosyringone), resuspended in infiltration media and diluted to a final OD_600_ of 0.02. The underside of the leaf to be infiltrated was punctured by a 200 μl pipette tip and up to 0.5 ml of agrobacterium solution was infiltrated in the leaf using a 1 ml syringe. After two days 1–2 cm^2^ leaf segments of the infiltrated area were excised and analysed by confocal laser scanning microscopy (LSM 510, AxioObserver Carl Zeiss), using a *Plan-Apochromat 20x/0.8 M27* objective. Yellow fluorescent protein fluorescence was monitored with a 519–612 nm band pass emission filter (514 nm excitation). Images were processed using ZEN2011 software (Carl Zeiss).

## Figures and Tables

**Table 1:** SvPAL protein properties. The kinetic parameters (±standard deviation), with l-phenylalanine as substrate, were calculated from three separate measurements with three technical repeats each.

Isozyme	SvPAL1	SvPAL2	SvPAL3	SvPAL4
CDS length	2160	2136	2148	2136
Exon	2	2	2	2
Intron length	741	1088	794	709
aa length	716	711	715	711
Predicted molecular weight (Da)	77.885	77.473	77.842	77.659
pI	6.56	6.43	6.03	6.19
Enzyme kinetics done at pH	8.8	8.8	9.0	
Kinetics				
*K_m_* (μM)	81.6 ± 0.30	32.35 ± 0.50	88.41 ± 0.55	
*V*_max_ (pkat/μg protein)	15.5 ± 0.43	7.13 ± 0.43	27.16 ± 1.32	
*k*_cat_ (s^−1^)	22.79	8.35	28.98	
*k*_cat_/*K_m_* (s^−1^ M^−1^)	279,495	258,242	327,748	

**Table 2:** List of primer sequences used for cloning and semi-qRT-PCR.

Name	Cloning/qPCR	Primer sequence (5′ → 3′)
SvPAL1-qPCR-Forward	qPCR	GGCTCTTGTCAATGGAACAGCAGTT
SvPAL1-qPCR-Reverse	qPCR	GGCGAAAATTGCAGAAATGAGTTCT
SvPAL2-qPCR-Forward	qPCR	CGAGGTGAAGCGCATGGTTGAC
SvPAL2-qPCR-Reverse	qPCR	ACTCGAGCCTCCTCCGACAA
SvPAL3-qPCR-Forward	qPCR	TGCTCTGGTTAATGGAACTGCAGTC
SvPAL3-qPCR-Reverse	qPCR	AGCAAAAATTGCCGACAAGAGCTCC
SvPAL4-qPCR-Forward	qPCR	TGAGGTCAAGCGAATGATCGAG
SvPAL4-qPCR-Reverse	qPCR	GCTCGAGCCTCCTCTGACAG
SvTIP4-like-F	qPCR	CGATCGAATCCGTAATTAAAATTCC
SvTIP4-like-R	qPCR	CGGCTTTTAGGTCTTTGTCATCTAC
PAL1-TOPO-Forward	Cloning	CACCATGGAGACAATCACCAAGAATGGCTA
PAL1-TOPO-Reverse	Cloning	TCAACAGATTGGAAGAGGGGCG
PAL2-TOPO-Forward	Cloning	CACCATGGAATTCTGTCAGCACTCGAG
PAL2-TOPO-Reverse	Cloning	TCAACAAAGAGGAAGAGGAGCAC
PAL3-TOPO-Forward	Cloning	CACCATGGCCACCAAAATGGCTCT
PAL3-TOPO-Reverse	Cloning	TTAACAGATAGGAAGAGGGGAACCATT
PAL4-TOPO-Forward	Cloning	CACCATGGAATCCTGTCAAGATTCACGC
PAL4-TOPO-Reverse	Cloning	TTAGCAAATAGGAAGAGGAGCACCA
PAL1-ns-TOPO-Forward	Cloning	CACCATGGAGACAATCACCAAGAATGGCTA
PAL1-ns-TOPO-Reverse	Cloning	ACAGATTGGAAGAGGGGCG
PAL2-ns-TOPO-Forward	Cloning	CACCATGGAATTCTGTCAGCACTCGAG
PAL2-ns-TOPO-Reverse	Cloning	ACAAAGAGGAAGAGGAGCACC
PAL3-ns-TOPO-Forward	Cloning	CACCATGGCCACCAAAATGGCTCT
PAL3-ns-TOPO-Reverse	Cloning	ACAGATAGGAAGAGGGGAACCATT

## Appendix A Supplementary data

Supplementary Fig. S1 ClustalW alignment of *SvPAL* nucleotide sequences, with qPCR primer locations.

Supplementary Fig. S2 ClustalO alignment of SpPAL, SvPAL and PtrPAL.

Supplementary Fig. S3 Subcellular localization of SvPAL1 and 3.
