# Supplementary material for: Characterisation of the willow phenylalanine ammonia-lyase (PAL) gene family reveals expression differences compared with poplar
Source: Phytochemistry. 2015 Sep;117:90–7. doi: 10.1016/j.phytochem.2015.06.005 (PMC4560161; doi:10.1016/j.phytochem.2015.06.005)
Supplement: Supplementary Fig. S1 — ClustalW alignment of SvPAL nucleotide sequences, with qPCR primer locations. [file mmc1.pdf]

**Characterisation of the willow phenylalanine ammonia-lyase (*PAL*) gene family reveals expression differences compared with poplar.** Planta; Femke de Jong, Steve J. Hanley, Michael H. Beale, Angela Karp; Corresponding author: Femke de Jong, AgroEcology department, Rothamsted Research, Harpenden, Hertfordshire, AL5 2JQ, United Kingdom, femke.dejong@rothamsted.ac.uk.

```
SvPAL2          ATGGAATT--CTGTCAGGACTCGA----GCAATGGCAATGGTTTCCT--GGGATTCA-AC 51
SvPAL4          ATGGAATC--CTGTCAAGATTCAC----GCAATGGCAATGGTTCGCT--AGGGTTTA-AC 51
SvPAL1          ATGGAGACAATCACCAAGAATGGCTACCAAAATGGCGGTTCTTTGTCGGAGAGTTTGTGC 60
SvPAL3          ATGGAGACAGTCACCAGGAATGGCCACCAAAATGGC-----TCTG-CGGAGAGTTTGTGC 54
                *****         ** ** *         ******     *        *  **    *

SvPAL2          CCCAG------------TGACCCCTTGAACTGGGGCATGGTGGCAGAGTCGCTAAAAGGG 99
SvPAL4          ACAAA------------TGACCCTTTGAACTGGGGCATGGCGGCAGAATCGTTAAAAGGA 99
SvPAL1          ACCAGTAACCAGCACGGTGATCCCCTCAGCTGGGGTGTTGCCGCTGATGCAATGAAAGGA 120
SvPAL3          ACCA---AACAACA---TGATCCGCTCAGCTGGGGAGTTGCTGCTGAGGCAATGAAAGGC 108
                 * *             *** **  * * ******  * *  ** **  *  * *****

SvPAL2          AGCCACCTGGACGAGGTGAAGCGCATGGTTGACGAGTACAGGAAGCCCGTGGTCAAGTTA 159
SvPAL4          AGCCACCTAGATGAGGTCAAGCGAATGATCGAGGAGTACAGGAAACCTGTGGTCAAGTTA 159
SvPAL1          AGCCATTTGGATGAAGTGAAACGTATGGTTGCTGAGTACAGGAAGCCTGCTGTGAATCTC 180
SvPAL3          AGCCACTTGGATGAAGTGAAGCGTATGGTGGCTGATTATAGGAAGCCTGTTGTCAAGCTT 168
                *****  * ** ** ** ** ** *** * *  ** ** ***** ** *  ** **  *

SvPAL2          GGAGGTGCGACCCTGACCATAGGCCAAGTAGCAGCCATTGCTAGCCGTGATGTTGG---T 216
SvPAL4          GGAGGTGACACCCTGACCATAGGCCAAGTAGCTGCAATCGCTAGCCGTGACGTTGG---T 216
SvPAL1          GGTGGCCAGACCTTGACCATTGCTCAGGTGGCGTCTATTGCCGCTCACGATGCTAGCAGT 240
SvPAL3          GGAGGGGAGACCCTGACCATTGCTCAGGTCGCATCCATTGCCGGTCACGATGCCGGCGAT 228
                ** **    *** ******* *  ** ** **  * ** **    *  ** *   *   *

SvPAL2          GTCAAGGTGGAGTTGTCGGAGGAGGCTCGAGTCGGTGTCAAGGCTAGCAGTGACTGGGTT 276
SvPAL4          GTCAGGGTAGAGCTGTCAGAGGAGGCTCGAGCCGGTGTCCAAGCCAGCAGTGACTGGGTC 276
SvPAL1          GTTAAGGTGGAGCTTTCCGAGTCTGCCAGACCTCGCGTCAAGGCTAGCAGCGACTGGGTC 300
SvPAL3          GTCACGGTGGAGCTTTCCGAGTCTGCAAGAGCTGGTGTCAAGGCTAGCAGTGACTGGGTC 288
                ** * *** *** * ** ***   **  **    * *** * ** ***** ********

SvPAL2          ATGGACAGCATGAGCAAGGGCACGGACAGTTACGGTGTCACCACTGGTTTCGGTGCAACC 336
SvPAL4          ATGGACAGTATGAATAAGGGCACAGACAGTTATGGCGTGACTACTGGTTTTGGTGCAAAC 336
SvPAL1          ATGGATAGCATGGGCAAGGGAACCGATAGCTATGGTGTCACTACTGGGTTTGGTGCTACT 360
SvPAL3          ATGGATAGCATGGACAAGGGAACGGATAGCTACGGTGTTACTACTGGTTTTGGTGCCACT 348
                ***** ** ***   ***** ** ** ** ** ** ** ** ***** ** ***** *

SvPAL2          TCACATAGAAGAACCAAGCAGGGTGGAGAGCTTCAGAAGGAGCTTATTAGGTTCTTGAAT 396
SvPAL4          TCACATAGAAGAACCAAGCAAGGTGGAGAGCTCCAGAAGGAGCTTATTAGGTTTTTGAAT 396
SvPAL1          TCCCACAGACGAACCAAACAGGGCGGTGCTCTTCAGAAGGAACTCATTCGATTCTTGAAT 420
SvPAL3          TCTCACAGAAGAACCAAACAAGGAGGTGCTCTTCAGAGCGAACTCATTAGATTCTTGAAT 408
                ** ** *** ******* ** ** ** *  ** ****  ** ** *** * ** ******

SvPAL2          GCTGGTATCTTTGGGAATGGCACAGAGTCTACTCATACGTTGCCTCACTCGGCATCCAGG 456
SvPAL4          GCTGGTATCTTTGGCAATGGCACGGAGTCAAGCCACACATTGCCTCACTCAGCAACCAGG 456
SvPAL1          GCTGGGATCTTTGGCAATGGAACAGAGACTTGCCACACACTGCCTCACCCAGCAACTAGA 480
SvPAL3          GCTGGGATCTTCGGAAATGGAACGGAAACTTCCCACACACTGCCTCACTCAGCAACTAGA 468
                ***** ***** ** ***** ** **  *    ** **  ******** * *** * **

SvPAL2          GCAGCCATGTTGGTCAGAATTAACACCCTTCTTCAAGGTTATTCWGGTATAAGATTTGAG 516
SvPAL4          GCAGCTATTTTGGTAAGAATCAACACCCTGCTTCAAGGTTACTCGGGCATAAGATTTGAA 516
SvPAL1          GCAGCCATGCTTGTCAGGATCAACACTCTCCTCCAGGGTTACTCTGGCATCAGGTTTGAA 540
SvPAL3          GCAGCCATGCTTGTCAGAATCAACACTCTCCTCCAAGGTTACTCTGGCATCAGATTTGAA 528
                ***** **  * ** ** ** ***** ** ** ** ***** ** ** ** ** *****

SvPAL2          ATMTTGGAAGCCATCACCAAGTTGCTCAACCACAACATCACCCCATGTTTGCCACTCAGA 576
SvPAL4          ATATTGGAAGCTATGAGCAAGTTGCTCAACCACAACATTACCCCATGCTTGCCACTCAGA 576
SvPAL1          ATCTTGGAGGCAATTACCAAGCTGCTTAACAACAACGTCACTCCATGCTTGCCACTACGT 600
SvPAL3          ATCTTGGAAGCCATTACCAAGCTACTCAATAACAACGTCACTCCATGCTTGCCACTACGA 588
                ** ***** ** ** * **** * ** **  ***** * ** ***** ********  *

SvPAL2          GGAACCATCACTGCGTCTGGTGACCTCGTCCCGTTATCTTACATTGCCGGGCTCCTGACA 636
SvPAL4          GGAACAATCACTGCCTCTGGTGACCTTGTCCCATTGTCTTATATTGCTGGGCTCTTGACA 636
SvPAL1          GGCACAATCACTGCTTCAGGTGATTTGGTCCCGTTATCCTACATTGCCGGGTTGCTGACC 660
SvPAL3          GGCACGATCACTGCTTCAGGTGATTTAGTCCCACTATCCTACATTGCTGGATTGCTGACT 648
                ** ** ******** ** *****  * *****  * ** ** ***** **  *  ****
```

```
SvPAL2          GGCAGACCCAATTCAAAGGCTGTGGGGCCCAATGGAGAACCCCTGGCCGCCGTTGAAGCC 696
SvPAL4          GGCAGGCCCAATTCAAAGGCAACTGGGCCCGATGGAGAGCCCCTGGGCCCTGTTGAAGCC 696
SvPAL1          GGCCGGCCTAATTCCAAGGCCACTGGTCCCAACGGAGAAGTGCTAGATGCTGCTGAGGCC 720
SvPAL3          GGCAGGCTCAATTCCAAGGCCACTGGCCCCAACGGAGAAGTGCTAGGTGCTGATGAAGCA 708
                *** * *  ***** *****    ** *** * *****    ** *   * * *** **

SvPAL2          TTTACACTAGCTGGGATCAATGGTGGATTCTTTGAGTTGCAGCCGAAGGAGGGGCTAGCA 756
SvPAL4          TTTGAACTAGCTGGGATTGATGGTGGTTTTTTTGAGCTGCGTCCAAAGGAGGGTTTTGCA 756
SvPAL1          TTTAAAGCAGCTGGTATTGATTCTGGGTTCTTCGAGTTGCAGCCTAAGGAAGGCCTGGCT 780
SvPAL3          TTCAAAGCAGCTGGTATTGAATCTGGATTCTTCGAGTTGCAGCCTAAAGAAGGCCTTGCT 768
                **   *  ****** **  *   *** ** ** *** ***  ** ** ** **  * **

SvPAL2          CTTGTAAATGGCACTGCTGTTGGTTCTGGTCTTGCTTCAATGGTGCTTTTTGAGACTAAT 816
SvPAL4          CTAGTAAATAGTACTGCTGTGGGTTCTGGTCTAGCTTCATTGGTGCTTTTTGAGGCTAAT 816
SvPAL1          CTTGTCAATGGAACAGCAGTTGGTTCTGGCTTGGCTTCCATGGTTCTTTTCGAGACCAAC 840
SvPAL3          CTGGTTAATGGAACTGCAGTCGGTTCCGGCTTGGCTTCTATGGTTCTTTTCGAGACCAAC 828
                ** ** *** * ** ** ** ***** **  * *****  **** ***** *** * **

SvPAL2          GTGTTGGCAATCTTATCAGAAGTTCTGTCGGCAATTTTCGCTGAAGTTATGCAAGGGAAA 876
SvPAL4          GTGCTTGCAATCCTATCAGAAGTTTTGTCAGCAGTTTTTGCCGAAGTTATGCAAGGAAAA 876
SvPAL1          GTTCTTGCTGTCTTATCAGAACTCATTTCTGCAATTTTCGCCGAGGTTATGAATGGTAAA 900
SvPAL3          GTTCTTGCTGTCTTATCGGAGCTCTTGTCGGCAATTTTTGCTGAGGTTATGAATGGTAAA 888
                **  * **  ** **** **  *  * ** *** **** ** ** ****** * ** ***

SvPAL2          CCAGAGTTTACTGATCACCTGACACATAAATTGAAGCATCATCCTGGCCAAATCGAAGCG 936
SvPAL4          CCCGAGTTTACTGATCACTTGACACATAAATTAAAGCATCATCCTGGCCAAATTGAAGCT 936
SvPAL1          CCTGAATTCACTGACCATCTGACACACAAATTGAAGCACCATCCAGGTCAGATTGAGGCT 960
SvPAL3          CCCGAATTCACCGACCATCTGACACACAAGTTGAAGCACCATCCAGGTCAAATCGAGGCC 948
                ** ** ** ** ** **  ******* ** ** ***** ***** ** ** ** ** **

SvPAL2          GCAGCCATCATGGAACATATTTTAGTTGGTAGCTCTTACGTAAAAGAAGCTCAAAAGTTA 996
SvPAL4          GCAGCTATCATGGAACATATTTTAGATGGCAGCTTCTATGTTAAAGCAGCTCAAAAGTTA 996
SvPAL1          GCAGCTATAATGGAGCACATTTTAGATGGCAGCGCTTACATGAAGGCAGCCAAGAAGCTG 1020
SvPAL3          GCGGCTATAATGGAGCACATTTTAGATGGCAGCGCCTACATGAAGGCGGCTAAGAAGTTG 1008
                ** ** ** ***** ** ******* *** ***   **  * ** *  **  * *** *

SvPAL2          CATGAGATCGATCCTCTACAGAAGCCTAAACAAGACAGATATGCTCTCCGAACATCTCCT 1056
SvPAL4          CATGAGACCGATCAACTACAGAAACCCAGACAAGATCGATATGCTCTTCGAACATCTCCA 1056
SxPAL1          CATGAAATGGACCCCTTGCAGAAACCAAAGCAAGATCGCTATGCTCTGAGGACTTCACCC 1080
SvPAL3          CATGAAATGGACCCCTTGCAGAAACCAAAGCAGGATCGCTACGCTCTAAGGACTTCACCC 1068
                ***** *  ** *   * ***** ** *  ** **  * ** *****  * ** ** **

SvPAL2          CAATGGCTAGGTCCTCTCATTGAAGTGATTAGAACTTCAACAAAAATGATCGAACGAGAA 1116
SvPAL4          CAATGGCTAGGTCCTCTTATTGAAGTGATTAGAACCTCAACAAAAATGATTGAACGGGAA 1116
SvPAL1          CAATGGCTTGGCCCCCAGATTGAAGTGATCCGATTCTCAACCAAGTCAATTGAAAGAGAG 1140
SvPAL3          CAGTGGCTCGGTCCCCAGATTGAAGTCATCCGATTCTCGACCAAGTCAATTGAAAGAGAG 1128
                ** ***** ** ** *  ******** **  **   ** ** **    ** *** * **

SvPAL2          ATCAACTCTGTCAATGACAATCCATTGATTGATGTGTCAAGGAACAAGGCTCTCCATGGA 1176
SvPAL4          ATCAACTCTGTCAATGATAATCCATTGATTGACGTGTCAAGGAATAAGACTATAAATGGA 1176
SvPAL1          ATCAACTCGGTGAATGACAACCCTCTGATTGATGTTTCAAGGAACAAGGCCTTACATGGT 1200
SvPAL3          ATTAATTCAGTGAACGACAACCCTCTAATTGATGTTTCAAGGAACAAGGCTTTGCATGGG 1188
                ** ** ** ** ** ** ** **  * ***** ** ******** *** *  *  ****

SvPAL2          GGGAATTTCCAGGGGACTCCTATTGGTGTCTCCATGGACAACACTCGTTTGGCGATTGCT 1236
SvPAL4          GGGAATTTCCAGGGGACCCCTATTGGTGTTTCAATGGACAACACTCGTTTGGCCATTGCT 1236
SvPAL1          GGCAACTTCCAGGGAACTCCAATCGGAGTCTCAATGGACAATGTACGTCTGGCCATTGCA 1260
SvPAL3          GGTAACTTCCAGGGAACTCCAATCGGAGTCTCCATGGATAATGTGCGTCTGGCCATCGCA 1248
                ** ** ******** ** ** ** ** ** ** ***** **    *** **** ** **

SvPAL2          TCAATTGGCAAACTCATGTTTGCACAATTCTCGGAACTTGTTAATGACTACTACAACAAT 1296
SvPAL4          TCAATCGGCAAACTCATGTTTGCACAGTTCTCTGAACTTGTTAATGATTTTTACAACAAT 1296
SvPAL1          TCTATCGGCAAGCTCCTATTCGCTCAGTTCAGTGAGCTCGTGAATGATTTCTACAACAAT 1320
SvPAL3          GCAATAGGCAAGCTGCTATTTGCTCAGTTCAGTGAGCTCGTAAATGATTTCTACAACAAC 1308
                 * ** ***** **  * ** ** ** ***   ** ** ** ***** *  ********

SvPAL2          GGACTGCCTTCAAACCTCACCGGTGGCCGCAATCCAAGCTTGGATTACGGGTTCAAAGGT 1356
SvPAL4          GGATTGCCTTCAAACCTCACTGGTGGCCGCAATCCAAGCTTGGATTACGGGTTCAAAGGT 1356
SvPAL1          GGACTGCCATCAAATCTCACTGCTAGCAGAAATCCAAGTTTGGATTATGGTTTCAAAGGA 1380
SvPAL3          GGATTGCCATCCAATCTCACTGCTAGCAGGAATCCAAGCTTGGATTATGGCTTCAAGGGA 1368
                *** **** ** ** ***** * * ** * ******** ******** ** ***** **
```

```
SvPAL2          GCTGAAATTGCCATGGCATCTTACTGCTCAGAGCTCCAATTCCTTGCTAATCCTGTCACC 1416
SvPAL4          GCTGAAATTGCCATGGCATCTTACTGTTCAGAGCTCCAATTCCTTGCCAATCCTGTCACC 1416
SvPAL1          GCTGAAATAGCAATGGCCTCCTACTGTTCTGAGCTTCAGTATCTTGCTAATCCAGTCACC 1440
SvPAL3          GCTGAAATAGCAATGGCCTCCTACTGTTCCGAGCTTCAGTACCTCGCCAATCCAGTCACC 1428
                ******** ** ***** ** ***** ** ***** ** *  ** ** ***** ******

SvPAL2          AATCATGTCCAAAGCGCTGAACAACACAACCAAGATGTCAACTCCTTGGGATTGATTTCT 1476
SvPAL4          AATCATGTCCAGAGTGCTGAGCAACATAACCAAGATGTCAACTCCTTAGGCTTGATTTCT 1476
SvPAL1          AGCCATGTGCAAAGTGCCGAGCAGCACAATCAAGATGTTAACTCGTTGGGGCTTATTTCT 1500
SvPAL3          AGCCATGTGCAAAGTGCCGAGCAGCACAATCAAGATGTCAACTCCTTGGGGCTTATCTCT 1488
                *  ***** ** ** ** ** ** ** ** ******** ***** ** **  * ** ***

SvPAL2          GCAAGAAAAACGGCTGAAGCTGTTGACATATTGAAGCTCATGTCTACAACTTGGTTGGTT 1536
SvPAL4          TCGAGAAAAACAGCTGAAGCCGTCGACATATTGAAGCTCATGTCTACCACTTTCTTGGTT 1536
SvPAL1          TCAAGAAAGACAGCAGAAGCTGTCGATATCTTGAAGCTCATGTCCACAACTTTCTTAGTC 1560
SvPAL3          TCAAGAAAGACAGCAGAAGCCGTCGATATCTTGAAGCTCATGTCCACAACTTTCTTGGTG 1548
                 * ***** ** ** ***** ** ** ** ************** ** ****  ** **

SvPAL2          GCTCTTTGTCAGGCTATAGACTTGAGGCACATAGAGGAGAACCTGAAGAACACGGTGAAG 1596
SvPAL4          GGTCTATGCCAAGCCATTGACTTAAGGCATATAGAGGAGAACCTGAAGAACACGGTCAAG 1596
SvPAL1          GCACTCTGCCAAGCAGTTGACCTGAGGCATCTTGAGGAGAACCTGAGGAGCGCTGTCAAG 1620
SvPAL3          GCTCTTTGCCAAGCTATCGACTTGAGGCATTTGGAAGAGAACTTGAGGAGCGTGGTCAAG 1608
                *  ** ** ** **  * *** * *****  * ** ****** *** ** *   ** ***

SvPAL2          AGTGTTGTCAGCCAAGTTGCAAAGAGAGCCTTAACAATGGGCTTCAATGGCGAGCTTCAC 1656
SvPAL4          AACACTGTCAGCCAGGTTGCCAAGAGAGTCTTGACAATGGGCTTCAATGGAGAGCTTCAC 1656
SvPAL1          AACACTGTCAGTCAGGTATCCAAGAGGGTTTTAACTACAGGTCCCAACGGAGAACTCCAT 1680
SvPAL3          AACACAGTTAGCCAAGTATCCAAGAGGGTTTTAACTACAGGTGCCAATGGAGAACTTCAC 1668
                *     ** ** ** **  * ***** *  ** ** *  **   *** ** ** ** **

SvPAL2          CCCTCGAGATTCTGCGAGAAAGACTTGCTCAAGGTTGTCGACAGAGAACATGTCTTTGCC 1716
SvPAL4          CCTTCTAGATTATGTGAAAAAGACTTGCTTGAGGTTGTTGACAGAGAACATGTCTTTGCC 1716
SvPAL1          CCATCAAGGTTCTGTGAGAAGGAGCTGCTCAAGGTGGTTGATCGTGAATACGTCTTTGCT 1740
SvPAL3          CCGTCAAGGTTCTGTGAGAAGGAGTTGCTCAAGGTGGTTGATCGTGAATATGTGTTCGCG 1728
                ** ** ** ** ** ** ** **  ****  **** ** **  * *** * ** ** **

SvPAL2          TACATCGATGACCCTTGCAGTGCAACCTACCCATTAATGCAGAAACTAAGGCAAGTACTC 1776
SvPAL4          TACATCGACGATCCTTGCAGTGCAACCTATCCATTAATGCAAAAGCTAAGACAAGTACTA 1776
SvPAL1          TACGCCGATGACCCCTGCAGCGCCACCTATCCATTGATGCAGAAACTAAGGCAAGTTCTT 1800
SvPAL3          TATGTGGATGACCCCTGCAGTGCCACTTATCCATTGATGCAAAAACTAAGGCAAGTTTTT 1788
                **    ** ** ** ***** ** ** ** ***** ***** ** ***** *****  *

SvPAL2          GTTGAACATGCCCTGGTGAATGGTGAGAAGGAAAAGAATTCAAGCACCTCGATTTTCCAA 1836
SvPAL4          GTCGAGCATGCCTTGGTGAATGGCGAGAAGGAAAAGAATTCAACCACTTCGATTTTCCAA 1836
SvPAL1          GTTGACCATGCACTGGCAAATGGGGAGAATGAGAAGAGCGCAAGCACTTCAGTCTTCCAT 1860
SvPAL3          GTCGACCATGCATTGGAAAATGGAGAGAATGAGAAGAATTTGAGCACTTCAGTCTTCCAA 1848
                ** ** *****  ***  ***** ***** ** ****     * *** **  * *****

SvPAL2          AAGATTGGGGTTTTCGAGGAAGAACTGAAGACCCTTTTGCCGAAAGAAGTAGAGAGTGCT 1896
SvPAL4          AAGATCGGATCTTTCGAGGAAGAACTGAAGACCCTTTTACCTAAAGAGGTGGAGAGTGCC 1896
SvPAL1          AAGATCGGAGCTTTCGAGGAAGAGTTGAAGGCCGTTTTGCCGAAAGAAGTTGAGAGCGCA 1920
SvPAL3          AAGATTGAAGCTTTCGAGGAAGAATTGAAGGCTATTTTGCCGAAAGAAGTTGAGAGCGCC 1908
                ***** *    ************  ***** *  **** ** ***** ** ***** **

SvPAL2          AGACTTGAGATTGAGAACGGAAACCCATCTATTCCAAACAGGATCAAGGAATGCAGGTCA 1956
SvPAL4          AGACTTGAAGTCGAGAATGGAAATCCAGCTATCCCAAACAGGATCAACGAATGCAGGTCA 1956
SvPAL1          AGAACAGCATATGAGAGTGGGAACTCAGCAATTGAGAACAAGATTAAGGAATGCAGGTCT 1980
SvPAL3          AGAGCAGCGTATGATAGCGGGAATGCAGCCATTGACAACAAGATCAAGGAATGCAGGTCT 1968
                ***   *     ** *  ** **  ** * **    **** *** ** ***********

SvPAL2          TACCCCTTGTACAAGTTTGTGAGGGAAGAAGTGGGAACTAGTTTGCTTACTGGTGAGAAG 2016
SvPAL4          TACCCCTTGTACAAGTTTGTGAGGAAAGAACTGGGAACTAGTTTGCTAACAGGTGAGAAG 2016
SvPAL1          TATCCACTGTACAAGTTTGTGAGGGAGGAGTTGGGAACTGGCTTGCTCACCGGAGAAAAG 2040
SvPAL3          TATCCTCTGTACAAGTTTGTGAGGGAGGAGTTGGGAACTGAATTGCTCACCGGCGAAAAG 2028
                ** **  ***************** * **  ********   ***** ** ** ** ***

SvPAL2          GTCAGATCGCCCGGGGAGGAGTTTGACAAGGTATTCACAGCTATCTGTTCAGGGAAGTTG 2076
SvPAL4          GTGAAATCGCCTGGTGAGGATTTCGACAAGGTTTTCACAGCTATTTGTGCAGGGAAGCTG 2076
SvPAL1          GTCCAATCGCCCGGCGAGGAGTTCGACAAGGTGTTTACAGCTATGTGCCAGGGGAAGATC 2100
SvPAL3          GTCAAATCGCCTGGCGAGGAGTTCGACAGGGTGTTTACAGCAATGTGCCAGGGGAAGATC 2088
                **   ****** ** ***** ** **** *** ** ***** ** **    ****** *
```

```
SvPAL2          ATTGATCCCTTGCTGGAATGCTTGAAGGAATGGAATGGTGCTCCTCTTCCTCTTTGTTGA 2136
SvPAL4          ATTGGACCCTTGCTGGAATGCTTGAGGGAATGGAATGGTGCTCCTCTTCCTATTTGCTAA 2136
SvPAL1          ATTGACCCCATGCTGGAATGCCTTGGTGAATGGAACGGCGCCCCTCTTCCAATCTGTTGA 2160
SvPAL3          ATTGATCCAATGCTGGAATGCCTTGGGGAATGGAATGGTTCCCCTCTTCCTATCTGTTAA 2148
                ****  **  *********** *    ******** **  * ********  * ** * *
```

Supplementary Figure 1: ClustalW alignment of SvPAL nucleotide sequences. Identical bases are indicated by an asterisk (*) below the sequences. The primers for semi-qRT-PCR are underlined.
