# Supplementary material for: Characterisation of the willow phenylalanine ammonia-lyase (PAL) gene family reveals expression differences compared with poplar
Source: Phytochemistry. 2015 Sep;117:90–7. doi: 10.1016/j.phytochem.2015.06.005 (PMC4560161; doi:10.1016/j.phytochem.2015.06.005)
Supplement: Supplementary Fig. S2 — ClustalO alignment of SpPAL, SvPAL and PtrPAL. [file mmc2.pdf]

Characterisation of the willow phenylalanine ammonia-lyase (*PAL*) gene family reveals expression differences compared with poplar. Planta; Femke de Jong, Steve J. Hanley, Michael H. Beale, Angela Karp; Corresponding author: Femke de Jong, AgroEcology department, Rothamsted Research, Harpenden, Hertfordshire, AL5 2JQ, United Kingdom, femke.dejong@rothamsted.ac.uk.

```
PtrPAL3     METVTKNGYQNGSL--ESLCVN--QLDPLSWGVAAEAMKGSHLDEVKRMVADYRKPVVKL
SvPAL3      METVTRNGHQNGSA--ESLCTK--QHDPLSWGVAAEAMKGSHLDEVKRMVADYRKPVVKL
SpPAL3      -----------------MCTK--QHDPLSWGVAAEAMKGSHLDEVKRMVADYRKPVVKL
PtrPAL1     METITKNGYQNGSS--ESLCT---QRDPLSWGVAAEAMKGSHLDEVKRMVAEYRKPVVNL
SvPAL1      METITKNGYQNGGSLSESLCTSNQHGDPLSWGVAADAMKGSHLDEVKRMVAEYRKPAVNL
SpPAL1      METITKNGYQNG-SLSESLCTSNQHGDPLSWGVAADAMKGSHLDEVKRMVAEYRKPVVNL
PtrPAL2     MEFCQDSCTGNG-------SLGFNPNDPLNWGMVAESLKGSHLDEVKRMIDEYRKPVVKL
SpPAL2-1    MEFCQDSSNANG-------SLGFNPSDPLNWGMVAESLKGSHLDEVKRMVDEYRKPVVKL
SvPAL2      MEFCQDSSNGNG-------FLGFNPSDPLNWGMVAESLKGSHLDEVKRMVDEYRKPVVKL
SpPAL2-2    MEFCQDSSNANG-------SLGFNPSDPLNWGMVAESLKGSHLDEVKRMVDEYRMPVVKL
SvPAL4      MESCQDSRNGNG-------SLGFNTNDPLNWGMAAESLKGSHLDEVKRMIEEYRKPVVKL
SpPAL4      MEFCQDSRNGNG-------SLGFNTNDPLNWGMAAESLKGSHLDEVKRMIEEYRKPVVKL
PtrPAL4     MEFCQDSRNGNG-------SLGFNTNDPLNWGMAAESLKGSHLDEVKRMIEEYRKPVVKL
PtrPAL5     MEFCQDSRNGNG-------SLGFNTNDPLNWGMAAESLKGSHLDEVKRMIEEYRKPVVRL
                                      ***.**:.*:::***********: :** *.*.*
             N-Terminal domain                          MIO-domain

PtrPAL3     GGETLTIAQVASIAGHDTGDVKVELSESARPGVKASSDWVMDSMDKGTDSYGVTTGFGAT
SvPAL3      GGETLTIAQVASIAGHDAGDVTVELSESARAGVKASSDWVMDSMDKGTDSYGVTTGFGAT
SpPAL3      GGETLTIAQVASIAGHDAGDVTVELSESARAGVKASSDWVMDSMDKGTDSYGVTTGFGAT
PtrPAL1     AGQTLTIAQVASIAGHDASNVKVELSESARPRVKASSDWVMDSMDKGTDSYGVTTGFGAT
SvPAL1      GGQTLTIAQVASIAAHDASSVKVELSESARPRVKASSDWVMDSMGKGTDSYGVTTGFGAT
SpPAL1      GGQTLTIAQVASIAAHDDSSVKVELSESARPRVKASSDWVMDSMGKGTDSYGVTTGFGAT
PtrPAL2     GGESLTIGQVTAIASRDV-GVKVELSEEARVGVKASSDWVMDSMNKGTDSYGVTTGFGAT
SpPAL2-1    GGETLTIGQVAAIASRDV-GVKVELSEEARVGVKASSDWVMDSMSKGTDSYGVTTGFGAT
SvPAL2      GGATLTIGQVAAIASRDV-GVKVELSEEARVGVKASSDWVMDSMSKGTDSYGVTTGFGAT
SpPAL2-2    GGETLTIGQVAAIASRDV-GVKVELSEEARVGVKASSDWVMDSMSKGTDSYGVTTGFGAT
SvPAL4      GGDTLTIGQVAAIASRDV-GVRVELSEEARAGVQASSDWVMDSMNKGTDSYGVTTGFGAN
SpPAL4      GGETLTIGQVAAIASRDV-GVKVELSEEARAGVQASSDWVMDSMNKGTDSYGVTTGFGAN
PtrPAL4     GGETLTIGQVTAIASRDV-GVMVELSEEARAGVKASSDWVMDSMSKGTDSYGVTTGFGAT
PtrPAL5     GGETLTIGQVTAIASRDV-GVMVELSEEARAGVKASSDWVMDSMNS----HAVTAGFGAT
            .* :***.**::**.:*   * *****.**  *:********** .    :.**:****.
                                       MIO-domain

PtrPAL3     SHRRTKQGGALQKELIRFLNAGIFGNGTETCHTLPHSATRAAMLVRINTLLQGYSGIRFE
SvPAL3      SHRRTKQGGALQSELIRFLNAGIFGNGTETSHTLPHSATRAAMLVRINTLLQGYSGIRFE
SpPAL3      SHRRTKQGGALQRDLIRFLNAGIFGNGTETSHTLPHSATRAAMLVRINTLLQGYSGIRFE
PtrPAL1     SHRRTKQGGALQKELIRFLNAGIFGNGTETCHTLPHSATRAAMLVRINTLLQGYSGIRFE
SvPAL1      SHRRTKQGGALQKELIRFLNAGIFGNGTETCHTLPHPATRAAMLVRINTLLQGYSGIRFE
SpPAL1      SHRRTKQGGALQKELIRFLNAGIFGNGTETCHTLPHPATRAAMLVRINTLLQGYSGIRFE
PtrPAL2     SHRRTKQGGELQKELIRFLNAGIFGNGTESHTLPHSASRAAMLVRINTLLQGYSGIRFE
SpPAL2-1    SHRRTKQGGELQKELIRFLNAGIFGNGTESTHTLPHSASRAAMLVRINTLLQGYSGIRFE
SvPAL2      SHRRTKQGGELQKELIRFLNAGIFGNGTESTHTLPHSASRAAMLVRINTLLQGYSGIRFE
SpPAL2-2    SHRRTKQGGELQKELIRFLNAGIFGNGTESTHTLPHSASRAAMLVRINTLLQGYSGIRFE
SvPAL4      SHRRTKQGGELQKELIRFLNAGIFGNGTESSHTLPHSATRAAILVRINTLLQGYSGIRFE
SpPAL4      SHRRTNQGGELQKELIRFLNAGIFGNGTESSHTLPHSATRAAMLVRINTLLQGYSGIRFE
PtrPAL4     SHRRTKQGGELQKELIRFLNAGIFGNGTESSHTLPRSATRAAMLVRINTLLQGYSGIRFE
PtrPAL5     SHRRTKQGGELQKELIRFLNAGIFGNGTESSHTLPCSATRAAMLVRTNTLLQGYSGIRFE
            *****:*** ** :***************: ****  *:***:*** *************
                                  MIO-domain

PtrPAL3     ILEAITRLLNNNITPCLPLRGTITASGDLVPLSYIAGLLTGRPNSKATGPTGEVLDAAEA
SvPAL3      ILEAITKLLNNNVTPCLPLRGTITASGDLVPLSYIAGLLTGRLNSKATGPNGEVLGADEA
SpPAL3      ILEAITKLLNNNVTPCLPLRGTITASGDLVPLSYIAGLLTGRLNSKATGPNGEVLGADEA
PtrPAL1     ILEAITKLLNNNITPCLPLRGTITASGDLVPLSYIAGLLTGRPNSKATGPNGEVLDAVEA
SvPAL1      ILEAITKLLNNNVTPCLPLRGTITASGDLVPLSYIAGLLTGRPNSKATGPNGEVLDAAEA
SpPAL1      ILEAITKLLNNNVTPCLPLRGTITASGDLVPLSYIAGLLTGRPNSKATGPNGEVLDAAEA
PtrPAL2     ILEAITKLLNHNITPCLPLRGTITASGDLVPLSYIAGLLTGRPNSKAVGPNGETLAAAEA
SpPAL2-1    ILEAITKLLNHNITPCLPLRGTITASGDLVPLSYIAGLLTGRPNSKAVGPNGEPLAAVEA
SvPAL2      ILEAITKLLNHNITPCLPLRGTITASGDLVPLSYIAGLLTGRPNSKAVGPNGEPLAAVEA
SpPAL2-2    ILEAITKLLNHNITPCLPLRGTITASGDLVPLSYIAGLLTGRPNSKAVGPNGEPLSAVEA
SvPAL4      ILEAMSKLLNHNITPCLPLRGTITASGDLVPLSYIAGLLTGRPNSKATGPDGEPLGPVEA
SpPAL4      ILEAMSKLLNHNITPCLPLRGTITASGDLVPLSYIAGLLTGRPNSKATGPDGEPLGPVEA
PtrPAL4     MLEAITKLLNHNITPCLPLRGTITASGDLVPLSYIAGLLTGRPNSKAVGPNGEPLSPAEA
PtrPAL5     MLEAITKLLNHNITPCLPLRGTITASGDLVPLSYIAGLLTGRPNSKAVGPNGEPLSPAEA
            :***:::***.*:***************************** ****.** ** *   **
                                       MIO-domain
```

```
PtrPAL3      FKAAGIESGFFELQPKEGLALVNGTAVGSGLASMVLFETNVLAVLSELLSAIFAEVMNGK
SvPAL3       FKAAGIESGFFELQPKEGLALVNGTAVGSGLASMVLFETNVLAVLSELLSAIFAEVMNGK
SpPAL3       FKAAGIESGFFELQPKEGLALVNGTAVGSGLASMVLFETNVLAVLSELLSAIFAEVMNGK
PtrPAL1      FKAAGIDSGFFELQPKEGLALVNGTAVGSGLASMVLFETNVLAVLSELISAIFAEVMNGK
SvPAL1       FKAAGIDSGFFELQPKEGLALVNGTAVGSGLASMVLFETNVLAVLSELISAIFAEVMNGK
SpPAL1       FKAAGIDSGFFELQPKEGLALVNGTAVGSGLASMVLFETNVLAVLSELISAIFAEVMNGK
PtrPAL2      FTLAGINGGFFELQPKEGLALVNGTAVGSGLASMVLFEANVLAILSEVLSAIFAEVMQGK
SpPAL2-1     FTLAGINGGFFELQPNEGLALVNGTAVGSGLASMVLFEANVLAILSEVLSAIFAEVMQGK
SvPAL2       FTLAGINGGFFELQPKEGLALVNGTAVGSGLASMVLFETNVLAILSEVLSAIFAEVMQGK
SpPAL2-2     FTLAGINGGFFELQPKEGLALVNGTAVGSGLASMVLFETNVLAILSEVLSAIFAEVMQGK
SvPAL4       FELAGIDGGFFELRPKEGFALVNSTAVGSGLASLVLFEANVLAILSEVLSAVFAEVMQGK
SpPAL4       FELAGIDGGFFELHPKEGFALVNSTAVGSGLASLVLFEANVLAIISEVLSAVFAEVMQGK
PtrPAL4      FTQAGIDGGFFELQPKEGLALVNGTAVGSGLASMVLFETNVLAILSEVLSAIFAEVMQGK
PtrPAL5      FTQAGIDGGFFELQPKEGLALVNGTAVGSGLASMVLFETNVLAILSEVLSAIFAEVMQGK
             *  ***:.*****:*:**:****.*********:****:****::**::**:*****:**
                   MIO-domain                        Core-domain

PtrPAL3      PEFTDHLTHKLKHHPGQIEAAAIMEHILDGSAYMKAAKKLHETDPLQKPKQDRYALRTSP
SvPAL3       PEFTDHLTHKLKHHPGQIEAAAIMEHILDGSAYMKAAKKLHEMDPLQKPKQDRYALRTSP
SpPAL3       PEFTDHLTHKLKHHPGQIEAAAIMEHILDGSAYMKAAKKLHEMDPLQKPKQDRYALRTSP
PtrPAL1      PEFTDHLTHKLKHHPGQIEAAAIMEHILDGSAYMKAAKKLHEMDPLQKPKQDRYALRTSP
SvPAL1       PEFTDHLTHKLKHHPGQIEAAAIMEHILDGSAYMKAAKKLHEMDPLQKPKQDRYALRTSP
SpPAL1       PEFTDHLTHKLKHHPGQIEAAAIMEHILDGSAYMKAAKKLHEMDPLQKPKQDRYALRTSP
PtrPAL2      PEFTDHLTHKLKHHPGQIEAAAIMEHILDGSSYVKAAQKLHEIDPLQKPKQDRYALRTSP
SpPAL2-1     PEFTDHLTHKLKHHPGQIEAAAIMEHILVGSSYVKVAQKLHEIDPLQKPKQD*YALRTSP
SvPAL2       PEFTDHLTHKLKHHPGQIEAAAIMEHILVGSSYVKEAQKLHEIDPLQKPKQDRYALRTSP
SpPAL2-2     PEFTDHLTHKLKHHPGQIEAAAIMEHILVGSSYVKEAQKLHEIDPLQKPKQDRYALRTSP
SvPAL4       PEFTDHLTHKLKHHPGQIEAAAIMEHILDGSFYVKAAQKLHETDQLQKPRQDRYALRTSP
SpPAL4       PEFTDHLTHKLKHQPGQIEAAAIMEHILDGSSYVKAAQKLHETDPLQKPKQDRYALRTSP
PtrPAL4      PEFTDHLTHKLKHHPGQIEAAAIMEHILDGSAYVKEAQKLHEIDPLQKPKQDRYALRTSP
PtrPAL5      PEFTDHLTHRLKHHPGQIEAAAIMEHILDGSSYVKEAQKLNEIDPLQKPKKDRYALQTSP
             *********:***:************** ** *:* *:**.* * ****::* ***:***
                                   Core-domain

PtrPAL3      QWLGPQIEVIRFSTKSIEREINSVNDNPLIDVSRNKAIHGGNFQGTPIGVSMDNVRLAIA
SvPAL3       QWLGPQIEVIRFSTKSIEREINSVNDNPLIDVSRNKALHGGNFQGTPIGVSMDNVRLAIA
SpPAL3       HWLGPQIEVIRFSTKSIEREINSVNDNPLIDVSRNKALHGGNFQGTPIGVSMDNVRLAIA
PtrPAL1      QWLGPQIEVIRFSTKSIEREINSVNDNPLIDVSRNKALHGGNFQGTPIGVSMDNVRLAIA
SvPAL1       QWLGPQIEVIRFSTKSIEREINSVNDNPLIDVSRNKALHGGNFQGTPIGVSMDNVRLAIA
SpPAL1       QWLGPQIEVIRFSTKSIEREINSVNDNPLIDVSRNKALHGGNFQGTPIGVSMDNVRLAIA
PtrPAL2      QWLGPLIEVIRTSTKMIEREINSVNDNPLIDVSRNKALHGGNFQGTPIGVSMDNTRLAIA
SpPAL2-1     QWLGPLIEVIRTSTKMIEREINSVNDNPLIDVSRNKALHGGNFQGTPIGVSMDNTRLAIA
SvPAL2       QWLGPLIEVIRTSTKMIEREINSVNDNPLIDVSRNKALHGGNFQGTPIGVSMDNTRLAIA
SpPAL2-2     QWLGPLIEVIRTSTKMIEREINSVNDNPLIDVSRNKALHGGNFQGTPIGVSMDNTRLAIA
SvPAL4       QWLGPLIEVIRTSTKMIEREINSVNDNPLIDVSRNKTINGGNFQGTPIGVSMDNTRLAIA
SpPAL4       QWLGPLIEVIRTSTKKIEREINSVNDNPLIDVSRNKTLNGGNFQGTPIGVSMDNTRLAIA
PtrPAL4      QWLGPLIEVIRTSTKMIEREINSVNDNPLIDVSRNKALHGGNFQGTPIGVSMDNTRLAIA
PtrPAL5      QWLGPLIEVIRTSTKMIEREINSVNDNPLIDVSRNKALHGGNFQGTPIGVSMDNTRLAIA
             :**** ***** *** ********************::.***************.*****
                                   Core-domain

PtrPAL3      SIGKLLFAQFSELVNDFYNNGLPSNLTASRNPSLDYGFKGAEIAMASYCSELQYLANPVT
SvPAL3       AIGKLLFAQFSELVNDFYNNGLPSNLTASRNPSLDYGFKGAEIAMASYCSELQYLANPVT
SpPAL3       AIGKLLFAQFSELVNDFYNNGLPSNLTASRNPSLDYGFKGAEIAMASYCSELQFLANPVT
PtrPAL1      SIGKLLFAQFSELVNDFYNNGLPSNLTASRNPSLDYGFKGAEIAMASYCSELQYLANPVT
SvPAL1       SIGKLLFAQFSELVNDFYNNGLPSNLTASRNPSLDYGFKGAEIAMASYCSELQYLANPVT
SpPAL1       SIGKLLFAQFSELVNDFYNNGLPSNLTASRNPSLDYGFKGAEIAMASYCSELQYLANPVT
PtrPAL2      SIGKLMFAQFSELVNDYYNNGLPSNLTGGRNPSLDYGFKGAEIAMASYCSELQFLANPVT
SpPAL2-1     SIGKLMFAQFSELVNDYYNNGLPSNLTGGRNPSLDYGFKGAEIAMASYCSELQFLANPVT
SvPAL2       SIGKLMFAQFSELVNDYYNNGLPSNLTGGRNPSLDYGFKGAEIAMASYCSELQFLANPVT
SpPAL2-2     SIGKLMFAQFSELVNDYYNNGLPSNLTGGRNPSLDYGFKGAEIAMASYCSELQFLANPVT
SvPAL4       SIGKLMFAQFSELVNDFYNNGLPSNLTGGRNPSLDYGFKGAEIAMASYCSELQFLANPVT
SpPAL4       SIGKLMFAQFSELVNDFYNNGLPSNLTGGRNPSLDYGFKGAEIAMASYCSELQFLANPVT
PtrPAL4      SIGKLMFAQFSELVNDFYNNGLPSNLTGGRNPSLDYGFKGAEIAMASYCSELQFLANPVT
PtrPAL5      SIGKLMFAQFSELVNDFYNNGLPSNLTGGRNPSLDYGFKGAEIAMASYCSELQFLANPVT
             :****:**********:**********..************************:******
                                 Core-domain
```

```
PtrPAL3      THVQSAEQHNQDVNSLGLISSRKTAEAVDILKLMSTTFLVALCQAIDLRHLEENLKSAVK
SvPAL3       SHVQSAEQHNQDVNSLGLISSRKTAEAVDILKLMSTTFLVALCQAIDLRHLEENLRSVVK
SpPAL3       SHVQSAEQHNQDVNSLGLISSRKTAEAVDILKLMSTTFLVALCQAIDLRHLEENLRSVVK
PtrPAL1      SHVQSAEQHNQDVNSLGLISSRKTAESVDILKLMSTTFLVALCQAIDLRHLEENLRSAVK
SvPAL1       SHVQSAEQHNQDVNSLGLISSRKTAEAVDILKLMSTTFLVALCQAVDLRHLEENLRSAVK
SpPAL1       SHVQSAEQHNQDVNSLGLISSRKTAEAVDILKLMSTTFLVALCQAVDLRHLEENLRNAVK
PtrPAL2      NHVQSAEQHNQDVNSLGLISARKTAEAVEILKLMSTTWLVALCQAIDLRHIEENLKNTVK
SpPAL2-1     NHVQSAEQHNQDVNSLGLISARKTAEAVDILKLMSTTWLVALCQAIDLRHIEENLKNTVK
SvPAL2       NHVQSAEQHNQDVNSLGLISARKTAEAVDILKLMSTTWLVALCQAIDLRHIEENLKNTVK
SpPAL2-2     NHVQSAEQHNQDVNSLGLISARKTAEAVDILKLMSTTWLVALCQAIDLRHIEENLKNTVK
SvPAL4       NHVQSAEQHNQDVNSLGLISSRKTAEAVDILKLMSTTFLVGLCQAIDLRHIEENLKNTVK
SpPAL4       NHVQSAEQHNQDVNSLGLISSRKTAEAVDILKLMSTTFLVGLCQAIDLRHIEENLKNTVK
PtrPAL4      NHVQSAEQHNQDVNSLGLISSRKTAEAVDILKLMSTTFLVGLCQAVDLRHIEENLKSTVK
PtrPAL5      NHVQSAEQHNQDVNSLGLISSRKTAEAVDILKLMSTTFLVGLCQAVDLRHIEENLKSTVK
             .*******************:*****:*:********:**.****:****:****:..**
                           Core-domain                        Shielding

PtrPAL3      NTVSQVSKRVLTTGANGELHPSRFCEKELLKVVDREYVFAYVDDPCSATYPLMQKLRQVF
SvPAL3       NTVSQVSKRVLTTGANGELHPSRFCEKELLKVVDREYVFAYVDDPCSATYPLMQKLRQVF
SpPAL3       NTVSQVSKRVLTTGANGELHPSRFCEKELLKVVDREYVFAYVDDPCSATYPLMQKLRQVF
PtrPAL1      NTVSHVSKRVLTTGANGELHPSRFCEKELLKVVDREDVFAYADDPCSATYPLMQKLRQVL
SvPAL1       NTVSQVSKRVLTTGPNGELHPSRFCEKELLKVVDREYVFAYADDPCSATYPLMQKLRQVL
SpPAL1       NTVSQVSKRVLTTGANGELHPSRFCEKELLKVVDREYVFAYADDPCSATYPLMQKLRQVL
PtrPAL2      NTVSQVAKRVLTMGFNGELHPSRFCEKDLLKVVDREHAFTYIDDPCSATYPLMQKLRQVL
SpPAL2-1     SVVSQVAKRALTMGFNGELHPSRFCEKDLLKVVDREHVFAYIDDPCSATYPLMQKLRQVL
SvPAL2       SVVSQVAKRALTMGFNGELHPSRFCEKDLLKVVDREHVFAYIDDPCSATYPLMQKLRQVL
SpPAL2-2     SVVSQVAKRALTMGFNGELHPSRFCEKDLLKVVDREHVFAYIDDPCSATYPLMQKLRQVL
SvPAL4       NTVSQVAKRVLTMGFNGELHPSRLCEKDLLEVVDREHVFAYIDDPCSATYPLMQKLRQVL
SpPAL4       NTVSQVAKRVLTMGFNGELQPSRLCEKDLLEVVDREHVFAYIDDPCSATYPLMQKLRQVL
PtrPAL4      NTVSQVAKRVLTMGFNGELHPSRFCEKDLLKVVDREHVFSYIDDPCSATYPLMQNLRQVL
PtrPAL5      NTVSQVAKRVLAMGFSGELHPSRFCEKDLLKVVDREHVFSYIDDPCSATYPLMQKLRQVL
             ..**:*:**.*: * .***:***:***:**:***** .*:* ************:****:
                                       Shielding-domain

PtrPAL3      VDHALENGENEKNFSTSVFQKIEAFEEELKALLPKEVESARAAYDSGNSAIDNKIKECRS
SvPAL3       VDHALENGENEKNLSTSVFQKIEAFEEELKAILPKEVESARAAYDSGNAAIDNKIKECRS
SpPAL3       VDHALENGENEKNLSTSVFQKIEAFEEELKVILPKEVESARAAYDSGNAAIDNKIKECRS
PtrPAL1      VDHALANGENEKNTSTSVFQKITAFEEELKALLPKEVESARAAYDSGNSAIENKIKECRS
SvPAL1       VDHALANGENEKSASTSVFHKIGAFEEELKAVLPKEVESARTAYESGNSAIENKIKECRS
SpPAL1       VDHALANGENEKSASTSVFHKIGAFEEELKAVLPKEVESARAAYESGNSAIENKIKECRS
PtrPAL2      VDHALMNGEKEHNSSTSIFQKIGAFEEELKTLLPKEVENARLELENGNPAIPNRITECRS
SpPAL2-1     VEHALVNGEKEKNSSTSIFQKIGVFEEELKTLLPKEVESARLEIENGNPSIPNRIKECRS
SvPAL2       VEHALVNGEKEKNSSTSIFQKIGVFEEELKTLLPKEVESARLEIENGNPSIPNRIKECRS
SpPAL2-2     VEHALVNGEKEKNSSTSIFQKIGVFEEELKTLLPKEVESARLEIENGNPSIPNRIKECRS
SvPAL4       VEHALVNGEKEKNSTTSIFQKIGSFEEELKTLLPKEVESARLEVENGNPAIPNRINECRS
SpPAL4       VEHALVNGEKEKNSTTSIFQKIGSFEEELKTLLPKEVESARLEVENGNPAIPNRINECRS
PtrPAL4      VEHALVNGERERNSTTSIFQKIGSFEEELKTLLPKEVESARLEVENGNPAIPNRIKECRS
PtrPAL5      VEHALVNGERERNSTTTILQKIGSFEEELKTLLPKEVESARLEVENGNPAIPNRIKECRS
             *:*** ***.*:. :*::::**  ******.:******.**   :.** :* *:*.****
                           Shielding-domain                     C-terminal

PtrPAL3      YPLYKFVREELGTVLLTGEKVQSPGEEFDKVFTAMCQGKIIDPMLECLGEWNGSPLPIC
SvPAL3       YPLYKFVREELGTELLTGEKVKSPGEEFDRVFTAMCQGKIIDPMLECLGEWNGSPLPIC
SpPAL3       YPLYKFVREELGTVLLTGEKVKSPGEEFDRVFTAMCQGKIIDPMLECLGEWNGSPLPIC
PtrPAL1      YPLYKFVREELGTGLLTGEKVRSPGEEFDKVFTAMCQGKIIDPMLECLGEWNGAPLPIC
SvPAL1       YPLYKFVREELGTGLLTGEKVQSPGEEFDKVFTAMCQGKIIDPMLECLGEWNGAPLPIC
SpPAL1       YPLYKFVREELGTGLLTGEKVQSPGEEFDKVFTAMCQGKIIDPMLECLGEWNGAPLPIC
PtrPAL2      YPLYKFVREELGTILLTGEKVGSPGEEFDKVFTAICAGKLIDPMLECLKEWNGAPLPLC
SpPAL2-1     YPLYKFVREEVGTSLLTGEKVGSPGEEFDKVFTAICSGKLIDPLLECLKEWNGAPLPLC
SvPAL2       YPLYKFVREEVGTSLLTGEKVRSPGEEFDKVFTAICSGKLIDPLLECLNEWNGAPLPLC
SpPAL2-2     YPLYKFVREEVGTSLLTGEKVRSPGEEFDKVFTAICSGKLIDPLLECLNEWNGAPLPLC
SvPAL4       YPLYKFVRKELGTSLLTGEKVKSPGEDFDKVFTAICAGKLIGPLLECLREWNGAPLPIC
SpPAL4       YPLYKFVRKELGTSLLTGEKVKSPGEEFDKVFTAICAGKLIDPLLECLKEWNGAPLPNC
PtrPAL4      YPLYKFVREELGTSLLTGEKVKSPGEEFDKVFTAICAGKLIDPLLECLKEWDGAPLPIC
PtrPAL5      YPLYKFVREELGTSLLTGEKVKSPGEEFDKVFTAICAGKLIDPLLECLKEWDGAPLPIC
             ********:*:** ******* ****:**:****:* **:* *:**** **:*:*** *
                                       C-terminal domain
```

Supplementary Figure 2: ClustalO alignment of SpPAL, SvPAL and PtrPAL. Underlined are the five domains of the PAL proteins. The shaded amino acids are subsequently: the conserved Tyr110-loop, the active site (Ala-Ser-Gly), the stop codon in SvPAL2-1, and the phosphorylation site.
