# Supplementary material for: Characterisation of the willow phenylalanine ammonia-lyase (PAL) gene family reveals expression differences compared with poplar
Source: Phytochemistry. 2015 Sep;117:90–7. doi: 10.1016/j.phytochem.2015.06.005 (PMC4560161; doi:10.1016/j.phytochem.2015.06.005)
Supplement: Supplementary Fig. S3 — Subcellular localization of SvPAL1 and 3. [file mmc3.pdf]

Characterisation of the willow phenylalanine ammonia-lyase (PAL) gene family reveals expression differences compared with poplar. Planta; Femke de Jong, Steve J. Hanley, Michael H. Beale, Angela Karp; Corresponding author: Femke de Jong, AgroEcology department, Rothamsted Research, Harpenden, Hertfordshire, AL5 2JQ, United Kingdom, femke.dejong@rothamsted.ac.uk.

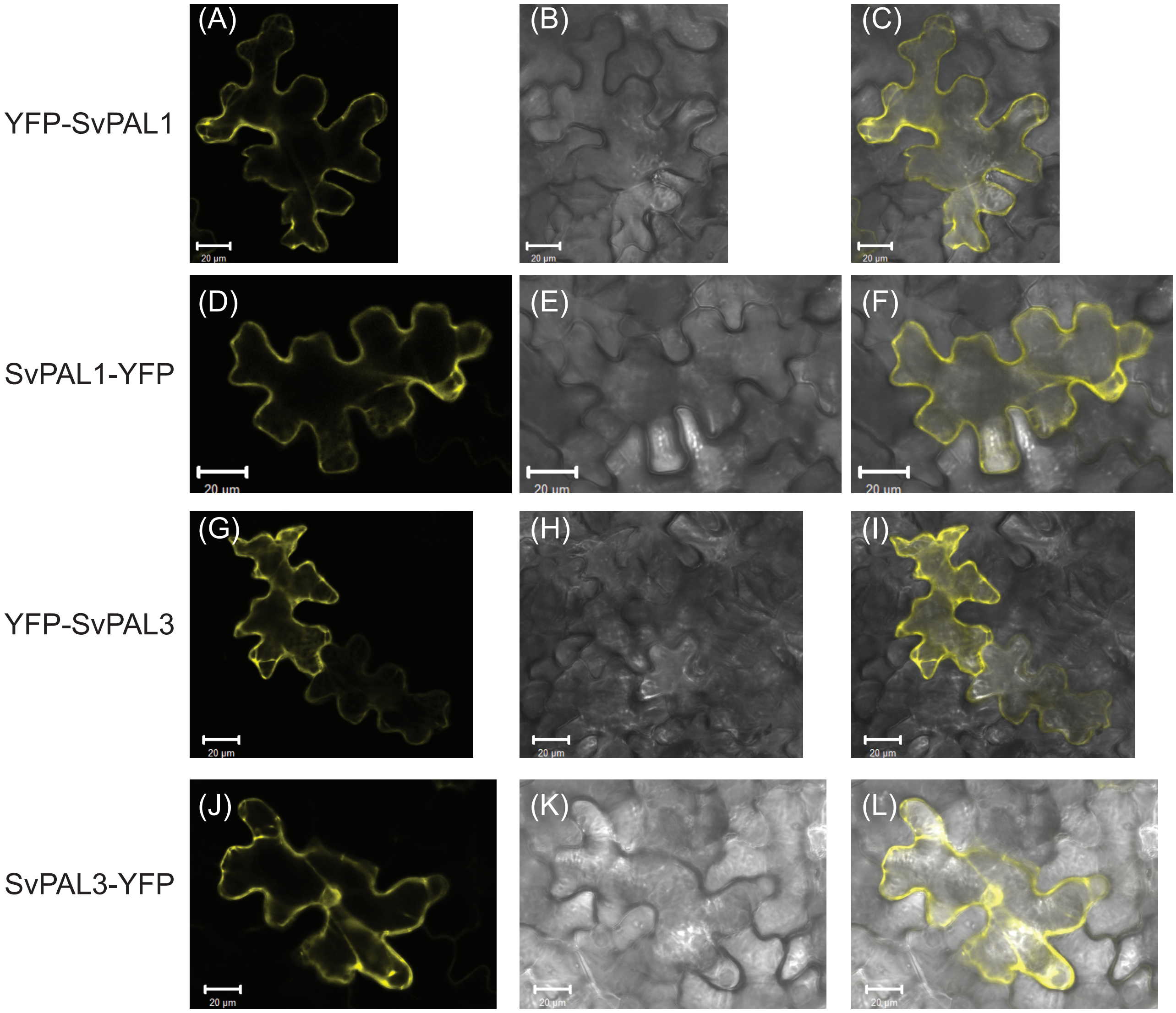

Supplementary Figure S3: Subcellular localization of SvPAL. An overview of the YFP fusion constructs is shown on the left, with the corresponding transient expression in tobacco epidermal cells is shown on the right. YFP fluorescence is shown in yellow (A, D, G, J). Transmission images of the tobacco epidermal cells are shown in the middle (B, E, H, K) and the overlay of the YFP fluorescence and the transmission images is shown at the far right (C, F, I, L). Bar is 20 µm.
